# PFKFB3 as a multifaceted driver and therapeutic target in castration-resistant prostate cancer

**DOI:** 10.1038/s41419-025-08089-8

**Published:** 2025-10-24

**Authors:** Lin Chen, Yu-Xin Xu, Ying-Ying Ren, Zhi-Da Wang, Xue-Man Dong, Yi-Min Chen, Pu Wu, Tong Wu, Fei Xiang, Tian Xie, Qi Zhang, Jian-Liang Zhou

**Affiliations:** 1 School of Pharmacy, Hangzhou Normal University, Hangzhou, Zhejiang China; 2 Zhejiang Provincial Key Laboratory of Anti-Cancer Chinese Medicines and Natural Medicines, Hangzhou Normal University, Hangzhou, Zhejiang China; 3 Department of Urology, Zhejiang Provincial People’s Hospital, Hangzhou, Zhejiang China

**Keywords:** Prostate cancer, Cell biology

## Abstract

Castration-resistant prostate cancer (CRPC) is the advanced stage of prostate cancer (PCa) progression, characterized by limited therapeutic options and significant challenges from drug resistance development. We show that PFKFB3, an essential regulator of glycolytic metabolism, is significantly upregulated in PCa tissues and CRPC cell lines, where it plays a pivotal role in driving CRPC progression. Knockdown of PFKFB3 or inhibition by a small molecule inhibitor significantly inhibits the growth and invasion of CRPC cells, whereas overexpression promotes malignant behaviors. Mechanistically, PFKFB3 modulates the PI3K/Akt-Wnt/β-catenin pathway, resulting in enhanced tumor cell proliferation. Additionally, combining a PFKFB3 inhibitor with docetaxel produces synergistic anti-CRPC effects and reduces toxicity. Therefore, PFKFB3-mediated metabolic reprogramming underlies CRPC progression, highlighting its potential as a therapeutic target and emphasizing the need for further exploration in the development of safe and effective PFKFB3 inhibitors for precise targeted therapy in CRPC.

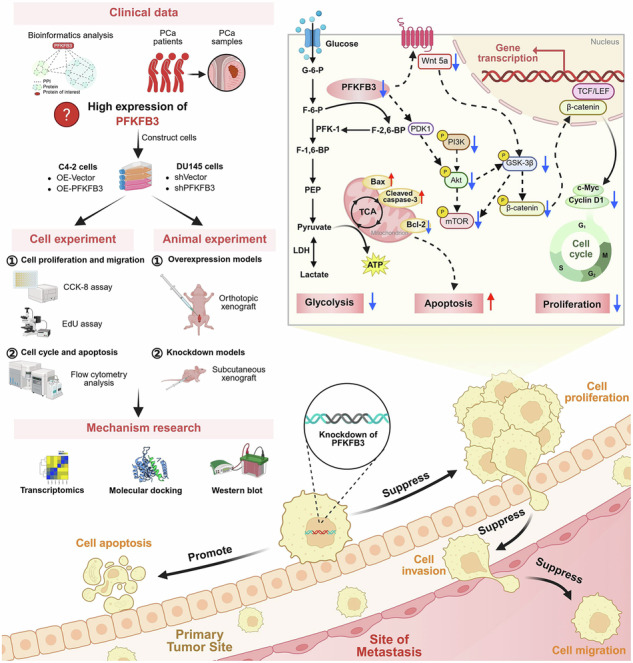

## Highlights

- Castration-resistant prostate cancer (CRPC) is marked by increased expression of PFKFB3, which promotes glycolytic reprogramming.
- PFKFB3 enhances tumor cell proliferation and migration via the PI3K/Akt-Wnt-catenin pathway.
- Silencing PFKFB3 reduces growth and invasion in CRPC, while its overexpression intensifies malignancy.
- Inhibiting PFKFB3 in conjunction with docetaxel (DTX) exhibits synergistic anti-tumor effects.

## Introduction

Prostate cancer (PCa) is the second most common malignancy among men globally and the fifth leading cause of cancer-related deaths [1, 2]. One of the most prevalent malignancies in men, PCa has significantly increased in incidence in China in recent years. Despite improvements in survival rates, the five-year survival rate for PCa remains significantly lower than in developed countries, highlighting a major clinical challenge [3]. For localized PCa, initial treatment options generally consist of radical prostatectomy, radiotherapy, and hormonal therapy, whereas androgen deprivation therapy (ADT) remains the cornerstone of treatment for advanced stages of PCa [4, 5]. A significant concern is that most patients progress to castration-resistant prostate cancer (CRPC) within 18 to 24 months of ADT, which is a key factor contributing to poor prognosis [6]. Endocrine therapy, chemotherapy such as docetaxel (DTX), and PI3K/Akt pathway inhibitors are the main treatment modalities for CRPC. However, associated damage to the skin, bone marrow, gastrointestinal system, and myocardium further compromises already weakened patients. Furthermore, the development of resistance during treatment significantly contributes to the limited clinical efficacy [7, 8]. Therefore, the development of safe and effective precision-targeted therapies, including poly ADP ribose polymerase inhibitors and immune checkpoint inhibitors, has become a key area of research for preventing and treating CRPC [9, 10].

In our prior research on the mechanism of action of the anticancer drug elemene against CRPC, we identified the major glycolytic enzyme phosphofructokinase-2/fructose-2,6-bisphosphatase 3 (PFKFB3) as a prominent regulatory target of elemene. Specifically, elemene downregulates PFKFB3 expression in CRPC cells by limiting its protein expression and promoting its degradation, resulting to the reduction of glycolysis in tumor cells. This suppression results in the inhibition of tumor cell proliferation and the promotion of apoptosis [11]. PFKFB3 is a bifunctional enzyme that plays a critical regulatory role in glycolysis by catalyzing the conversion of fructose-6-phosphate (F-6-P) to fructose-2,6-bisphosphate (F-2,6-BP), significantly increasing intracellular levels of F-2,6-BP. This increase activates phosphofructokinase-1, accelerating the glycolytic rate [12]. However, the role of other phosphofructokinase-2 (PFK-2) isoforms, such as PFKFB2 and PFKFB4, has also been recognized in PCa. Previous studies have identified PFKFB2 and PFKFB4 as key players in regulating glycolysis and tumor progression [13, 14]. In particular, these isoforms also catalyze the formation of F-2,6-BP, potentially influencing glycolytic flux and cancer metabolism. Studies indicate that PFKFB3 is highly expressed in various tumors, promoting rapid cell proliferation and tumor growth [15, 16]. In the tumor microenvironment, PFKFB3 overexpression (OE-PFKFB3) accelerates lactate production, lowers extracellular matrix pH, and enhances tumor cell invasiveness [17]. Furthermore, its association with tumor resistance and anti-apoptotic properties underscores the significance of PFKFB3 as an anticancer target [18]. Recently, PFKFB3 inhibitors have demonstrated efficacy in several cancers by effectively suppressing glycolytic metabolism, inhibiting tumor cell proliferation and survival [19]. However, although PFKFB3’s role has been reported in lung, nasopharyngeal, and gastric cancers, its function and potential clinical value in PCa remain unexplored [20–22]. Therefore, investigating the regulatory mechanisms of PFKFB3 in CRPC and its potential clinical value is essential for developing novel therapeutic strategies for CRPC.

This research explores the involvement of PFKFB3 in CRPC and assesses its potential as a therapeutic target. Bioinformatics analysis of data from The Cancer Genome Atlas (TCGA) suggests a strong association between glycolysis and PCa progression. Additional Venn diagram analysis and protein-protein interaction (PPI) network construction indicated that PFKFB3 is centrally involved in regulating the expression of glycolysis-related genes. To investigate the role of PFKFB3 in CRPC, we examined its impact on CRPC progression by manipulating PFKFB3 expression levels through knockdown and overexpression. The findings demonstrated that silencing PFKFB3 reduced the proliferation and invasion of CRPC cells in vitro, and markedly inhibited tumor growth in a CRPC mouse model. On the other hand, OE-PFKFB3 markedly accelerated the progression of CRPC. Further research revealed that PFKFB3 controls the progression of CRPC by effecting important signaling pathways, such as PI3K/Akt and Wnt/β-catenin, which are crucial for the metabolism and proliferation of cancer cells. To evaluate the potential of PFKFB3 as a therapeutic target for CRPC, we investigated the in vivo anti-CRPC activity of PFKFB3 inhibitor, the synergistic effect when combined with the first-line chemotherapy drug DTX, and the safety of PFKFB3 inhibitor alone and in combination with respect to organ damage and serum biochemical markers. The results demonstrated that the PFKFB3 inhibitor 3PO exhibited significant anti-CRPC efficacy and safety on its own and, when combined with DTX, produced synergistic anti-CRPC effects and reduced toxicity.

## Methods

### Cell culture

DU145, VCaP, and WPMY-1 cells were cultured in Dulbecco’s Modified Eagle Medium (Cat No. 11965092, Gibco) supplemented with 10% fetal bovine serum (FBS, Cat No. A5670701, Gibco). LNCaP, PC-3, 22RV-1 C4-2, and RM-1 cells were maintained in Roswell Park Memorial Institute-1640 Medium (RPMI-1640, Cat No. 11875119, Gibco) supplemented with 10% FBS. All cell lines were incubated at 37 °C in a humidified atmosphere with 95% air and 5% CO_2_.

### Clinical samples and experimental animals

Clinical PCa and benign prostatic hyperplasia (BPH) tissue samples were obtained from Zhejiang Provincial People’s Hospital, following approval from the hospital’s ethics committee (Ethics Approval No. KY2024124). All clinical samples were collected from patients undergoing prostatectomy. Written informed consent was obtained from patients before sampling. All experimental animals were kept in a 12-h light/dark cycle in the specified pathogen-free facility of Hangzhou Normal University. Prior to the study, five-week-old male BALB/c nude mice were used to establish subcutaneous PCa models. Mice were given free access to food and water and were allowed a one week adaptation period. The animal experiment protocol (Ethics Approval No. HSD20230303) was approved by the Animal Center of Hangzhou Normal University on March 10, 2023. All procedures adhered to the ethical standards for laboratory animal care set out by the National Institutes of Health.

### Subcutaneous PCa xenograft model

Five-week-old male BALB/c nude mice were acclimated for one week before being randomly assigned to two groups (*n* = 10 per group) based on body weight. Under sterile conditions, 5 × 10^6^ DU145 cells suspended in 100 μL phosphate-buffered saline (PBS) were subcutaneously injected into the left flank of each mouse to establish the subcutaneous xenograft model. Tumor size was measured every three days using a caliper, and tumor volume was calculated using the formula V = 0.5 × L × W^2^, where L represents the tumor’s longest diameter and W represents the shortest diameter. The experiment lasted for three weeks, after which mice were euthanized in accordance with ethical guidelines. Tumor tissues were harvested for subsequent analyses.

### Orthotopic PCa xenograft model

Five-week-old male BALB/c nude mice were acclimated for one week and then randomly divided into two groups (*n* = 6 per group) based on body weight. Under sterile conditions, mice were placed in a temperature-controlled environment and anesthetized with 1.5–2 Vol% isoflurane and an oxygen flow rate of 0.6–1 L/min. After anesthesia induction, mice were positioned on a heated surgical platform in a supine position. The surgical site was cleaned and disinfected under aseptic conditions. Using an insulin syringe, 5 × 10^5^ C4-2 cells suspended in a 25 μL PBS and Matrigel mixture (1:1 ratio) were precisely injected into the prostate. Following injection, the seminal vesicles and prostate were carefully repositioned, and the abdominal wall was sutured with absorbable stitches. Postoperatively, mice were housed individually with appropriate warming measures until full recovery. During the experiment, body weight was monitored every three days, and tumor volume was monitored weekly from day 7 post-surgery using an in vivo imaging system. The experiment lasted for three weeks, after which mice were euthanized, and tumor tissues were collected for further analysis. Animals found dead during the experiment were excluded from statistical analysis.

### In vivo small animal imaging (IVIS)

Orthotopic PCa xenograft model mice were injected with D-luciferin at a concentration of 10 μL/g body weight under isoflurane anesthesia. D-fluorescein is detected by a cold luminescence module without the need for excitation light. A corresponding volume of 15 mg/mL D-fluorescein solution was injected into the mouse, and images of the tumor area were subsequently captured using the IVIS system (PerkinElmer, model: Lumina XR). Tumor signal intensity was quantified by region of interest and statistical analysis was performed for each group of data.

### Drug toxicity screening study

The drug toxicity screening study was conducted using the subcutaneous xenograft model, following the same tumor induction procedure as described in the DU145 subcutaneous xenograft model. Once the tumor’s longest and shortest diameters reached approximately 6 mm, tumor-bearing mice were randomly assigned to four groups (*n* = 8 per group): control group, 3PO group, the DTX group, and a combination treatment group receiving both 3PO and DTX. Throughout the study, tumor volume were recorded every three days until the experiment concluded (lasting two weeks). Prior to euthanasia, mice were fasted for 12 h, followed by orbital blood collection and serum isolation via centrifugation. Finally, mice were euthanized via cervical dislocation, and major organs were excised, weighed, and subjected to further biochemical and pathological analyses.

### Data acquisition and differential expression analysis

The Prostate Adenocarcinoma (PRAD) dataset, which comprised 499 tumor samples and 52 normal controls, was made available by TCGA. To evaluate the variations in gene expression between PCa and normal tissues, the data was normalized and differential expression analysis was performed using R’s “limma” package (version 4.1.0). Genes that satisfied the significance thresholds of |log_2_ FC| > 0.585 and *P* < 0.05 were added to the enrichment analysis. Volcano plots and heatmaps, produced by the “ggplot2” and “pheatmap” R packages, respectively, were used to visualize gene distribution and expression in order to efficiently emphasize variations in expression.

### Gene set enrichment analysis (GSEA)

Gene expression profiles of tumor vs normal samples were graded using GSEA software (version 4.1.0, Broad Institute; https://www.gsea-msigdb.org/gsea/index.jsp) in order to evaluate the enrichment of particular gene sets, such as glycolysis-related genes from the MSigDB database. This research clarified important metabolic pathways involved in PCa and identified biological processes associated with disease characteristics. GSEA was performed with a significance level of *P* < 0.05, revealing information about the molecular mechanisms at play.

### Gene screening and PPI network analysis

Glycolysis-related genes with a relevance score higher than five were chosen from the GeneCards database and cross-referenced with differentially expressed genes from the TCGA dataset, which compares PCa and normal tissues. Important genes with important biological ramifications in PCa were identified by our investigation. The STRING database (https://string-db.org/) was then used to create PPI networks for these genes with a minimum confidence level of 0.40. The functional relationships between these important genes in PCa were then made clear by visualizing the PPI network using Cytoscape software (version 3.6.0, Cytoscape Consortium; https://cytoscape.org/).

### Hematoxylin and eosin (H&E) staining

H&E staining was used for histopathological examination of prostate tissue, as well as the heart, liver, spleen, lungs, and kidneys. Samples were fixed in 4% paraformaldehyde, dehydrated in graded ethanol, and embedded in paraffin. Paraffin-embedded tissues were sectioned at 4 μm thickness and then dewaxed. Sections were stained for five minutes with hematoxylin and rinsed with distilled water. Subsequently, sections were counterstained with eosin to visualize cytoplasm. After dehydration with 70% and 90% ethanol, followed by absolute ethanol, xylene was used for clearing. Lastly, neutral resin was used to mount the parts. Under an optical microscope (ECLIPSE Ts2, Nikon, Japan), tissue architecture and pathological alterations were examined and documented.

### Quantitative PCR (qPCR)

In accordance with the experimental design, cells were plated in 6-well plates at a density of 1 × 10^5^ cells per well and allowed to grow until 80% to 90% confluence was reached. For RNA extraction, cells were washed twice with PBS, and then 1 mL of TRIzol reagent was added to each well to lyse the cells. After lysis, 200 μL of chloroform was added to each well, mixed thoroughly, and incubated at room temperature for 3 min before centrifugation. The upper aqueous phase was collected, and isopropanol was added to precipitate the RNA. The RNA pellet was washed twice with 75% ethanol, air-dried, and resuspended in RNA dissolving solution to determine its concentration. For reverse transcription, the RNA was converted to cDNA using the Takara PrimeScript RT Reagent Kit following the manufacturer’s instructions. The qPCR was performed using SYBR Green Master Mix (Thermo Fisher), and relative gene expression was calculated using the 2^−ΔΔCT^ method, with GAPDH as the internal control. All experiments were performed in triplicate.

### Western blot analysis

After lysing the cells with freshly prepared RIPA buffer (1% NP-40, 0.1% SDS, and 1% sodium deoxycholate), the lysates were incubated on ice for 30 min. The protein concentrations were quantified using the BCA Protein Assay Kit (Pierce, USA). For each sample, 30 µg of protein was combined with loading buffer in equal proportions, heated at 95 °C for 5 min, and separated by SDS-PAGE at 80 V. After electrophoresis, the proteins were transferred to PVDF membranes at 110 V for 1.5 h. The membranes were then blocked with 5% non-fat milk at room temperature for 1 h. After blocking, the membranes were incubated with diluted primary antibodies at 4 °C overnight. On the following day, the membranes were washed with tris-buffered saline with Tween 20 (TBST) and incubated with secondary antibodies for 1 h at room temperature. Protein detection was performed using enhanced chemiluminescence (ECL) reagents (Thermo Fisher, USA), and images were captured on X-ray film. ImageJ software was used to evaluate relative protein expression, which was normalized to either GAPDH or β-actin.

### Lentivirus packaging and infection

Lentiviral vectors were used to achieve stable overexpression or knockdown of PFKFB3 in DU145 and C4-2 cells. For OE-PFKFB3, the pCDH-CMV-MCS-EF1-GFP+Puro vector was used, while for PFKFB3 knockdown (shPFKFB3), the pLKO.1-puro-CMV-GFP vector was employed. Corresponding empty vectors served as controls. Lentivirus production was performed in HEK293T cells via co-transfection with 6 µg of the transfer plasmid (OE-PFKFB3, shPFKFB3, or control vector), 4 µg of pPAX2, and 2 µg of pMD2.G (Addgene) using Lipofectamine 3000 (Thermo Fisher, USA). Viral supernatants were collected at 48 and 72 h post-transfection, followed by concentration using the Takara Bio Lenti-X Concentrator.DU145 and C4-2 cells were infected with lentivirus in the presence of 10 µg/mL polybrene for 24 h. Stable cell lines were established by selection with 2 µg/mL puromycin for seven days. Infection efficiency was validated by Western blot analysis and qPCR.

### Immunofluorescence

PCa cell samples grown on coverslips were fixed with 4% paraformaldehyde for 15 min at room temperature. After three PBS washes, the coverslips were permeabilized with 0.3% PBST (phosphate-buffered saline containing 0.1% Tween-20) for 15 min. Blocking was performed using 5% bovine serum albumin in PBST for 1 h at room temperature. The samples were then incubated overnight at 4 °C with a primary antibody against PFKFB3. Following PBS washes, a fluorescently labeled secondary antibody was applied and incubated in the dark for 1 h at room temperature. After additional PBS washes, the coverslips were mounted onto glass slides using an anti-fade mounting medium with DAPI for nuclear counterstaining. Fluorescence images were acquired using a confocal microscope and analyzed with ImageJ software.

### EdU staining assay

On coverslips, cells were seeded at a density of 1 × 10^4^ cells/well and grown to 80–90% confluence. After three PBS washes, cells were fixed with 4% paraformaldehyde for 15 min at room temperature. Following fixation, cells were permeabilized with 0.5% Triton ×-100 for 15 min, then rinsed with PBS. For EdU labeling, cells were incubated with 10 µM EdU reaction solution for 1 h at room temperature. After PBS washing, DAPI staining was applied for 5 min to counterstain the cell nuclei. The samples were mounted and observed under a fluorescence microscope (IX73, Olympus, Japan), where images were captured and analyzed.

### TUNEL staining

Cell coverslips were prepared as described for the TUNEL assay. Cells were seeded at a density of 1 × 10^5^ cells per well in 6-well plates and allowed to adhere overnight under standard conditions (37 °C, 5% CO_2_). Following treatment, cells were fixed with 4% paraformaldehyde for 30 min at room temperature. After fixation, cells were washed with PBS and permeabilized with 0.3% Triton ×-100 for 30 min. Following a PBS wash, the cells were incubated with TUNEL reaction buffer for 2 h at 37 °C in the dark. After incubation, cells were washed again with PBS, stained with DAPI for nuclear visualization, and mounted for fluorescence microscopy imaging.

### Cell proliferation and viability assay

Cell viability and proliferation were evaluated using the Cell Counting Kit-8 (CCK-8) assay. 5000 cells were plated per well in 96-well plates and incubated at 37 °C with 5% CO_2_ for 24 h to allow cell attachment. Treatment conditions were applied after the cells had adhered. For proliferation analysis, cells were treated with the respective compounds (drug concentrations specified in the relevant sections) for 24, 48, and 72 h. At each time point, 10 µL of CCK-8 reagent was added to each well, and the plates were incubated for an additional 2 h at 37 °C. Absorbance was measured at 450 nm using a microplate reader. Relative optical density (OD) values were calculated to assess cell proliferation and viability. Statistical analysis was performed using GraphPad Prism software (GraphPad Prism, GraphPad Software Inc., San Diego, CA, USA; https://www.graphpad.com/).

### Cell cycle analysis

After washing with PBS, cells were fixed overnight at 4 °C in 70% ethanol. Following fixation, cells were rinsed with PBS and resuspended in a staining buffer containing 50 µg/mL RNase and 50 µg/mL propidium iodide (PI). The cells were incubated at room temperature for 30 min. The cell cycle distribution was analyzed using the BD Accuri^TM^ C6 flow cytometer (BD Biosciences, San Jose, CA, USA), and the results were interpreted using ModFit LT software (Verity Software House, Topsham, ME, USA; https://www.vsh.com/products/modfit/).

### Apoptosis detection

Apoptosis was assessed using the FITC Annexin V Apoptosis Detection Kit. Cells were resuspended in 1 × Binding Buffer, followed by the addition of FITC Annexin V and PI. After a 15-min incubation at room temperature, apoptosis was measured using flow cytometry and the results were analyzed with FlowJo^TM^ software (FlowJo LLC, Ashland, OR, USA; https://www.flowjo.com/).

### Clonogenic assay

In 12-well plates, cells were plated at a density of 200 cells per well. After plating, the cells were allowed to incubate for 7 days under standard culture conditions. The cells were then fixed with 4% paraformaldehyde for 15 min and stained with 10% crystal violet solution for 30 min. The excess stain was washed off with PBS, and the colonies were allowed to air-dry. The colony formation was analyzed and quantified using ImageJ software.

### Scratch assay

Cells were cultivated until they reached 80–90% confluence. Serum-free media was added after 2 × 10^5^ cells were plated in 12-well plates and given a full day to adhere. The cell monolayer was scraped vertically using a 200 µL pipette tip. After removing the media and giving the cells two PBS washes, new serum-free medium was added. The healing rate was computed after 48 h of monitoring and photographing the closure of the scratch. Three duplicates of the experiment were conducted.

### Cell migration and invasion assays

Cells were plated in transwell chambers (8 µm pore size, Costar, USA) for the migration test. To promote cell migration, medium supplemented with 10% FBS was placed in the bottom chamber, and a cell suspension (5 × 10^4^ cells/100 µL) was placed in the top chamber. After a 24-h incubation at 37 °C with 5% CO_2_, the non-migrated cells on the membrane’s top surface were carefully removed using a cotton swab. The migrated cells were fixed using 4% paraformaldehyde for 15 min and then stained with crystal violet for 30 min. The photos were taken with an optical microscope (ECLIPSE Ts2, Nikon, Japan), and the number of migrated cells was quantified using ImageJ software. For the invasion experiment, transwell chambers were similarly pre-coated with Matrigel (BD Biosciences) and incubated at 37 °C for 30 min to allow the Matrigel to solidify. The rest of the procedure was identical to the migration assay.

### Transmission electron microscopy (TEM)

After being collected, cells were fixed for four hours at room temperature in 2.5% glutaraldehyde. Following fixation, cells were washed three times for five minutes each using 0.1 M PBS. After that, the cells were embedded in 1% agarose and fixed for two hours in 1% osmium tetroxide. A graded sequence of ethanol (30, 50, 70, 80, 95, and 100%) was used to dehydrate the mixture, and propylene oxide was then infused. Epoxy resin was then used to embed the samples. After cutting extremely thin sections, the materials were stained with lead citrate and uranyl acetate before being viewed and imaged using a transmission electron microscope (HT7800/HT7700, Hitachi, Japan).

### Reactive oxygen species (ROS) detection

After being grown to 80–90% confluence, the cells were carefully pipetted out in PBS. The cells were then treated with DCFH-DA at 37 °C for an hour. The cells were resuspended for fluorescence examination following a PBS rinse.

### Seahorse analysis

Cells (2 × 10^4^ cells/well) were seeded onto Cell-Tak-coated Seahorse XFe96 microplates (Agilent, USA) using a centrifugation protocol (200 × *g* for 1 min, zero brake) to ensure uniform adherence. After cell attachment, metabolic function was assessed using the glycolysis stress test and mitochondrial stress test kits according to the manufacturer’s instructions. For the glycolysis stress test, 10 mM glucose was first injected, followed sequentially by 2 µM oligomycin and 50 mM 2-deoxyglucose (2-DG). For the mitochondrial stress test, 2 µM oligomycin was initially injected, followed by 1 µM FCCP and a final injection of 0.5 µM rotenone/antimycin A. Oxygen consumption rate (OCR) and extracellular acidification rate (ECAR) were monitored throughout the assay using the Seahorse XFe96 Analyzer, and energy phenotype maps were generated by plotting OCR and ECAR values before and after oligomycin treatment.

### Fluorescence staining of organelles

Mito-Tracker Red CMXRos was applied to mitochondria for 30 min at 37 °C. Cells were then rinsed with PBS and counterstained with DAPI. A microscope (IX73, Olympus, Japan) was used for fluorescence imaging. Under the identical circumstances as for mitochondrial staining, ER-Tracker Green was used to stain the endoplasmic reticulum. Cells were treated with Golgi-Tracker Red for 30 min at 4 °C and then processed in the same way to visualize the Golgi apparatus. DAPI labeling and PBS washing were performed after cells containing the Lysotracker probe were incubated in culture media for two hours at 37 °C in a 5% CO_2_ atmosphere in order to identify lysosomes. After 20 min of room temperature incubation with a lipid droplet-specific dye, cells were exposed to PBS washing and DAPI labeling in order to label the lipid droplets. Every cellular structure was seen under a fluorescence microscope (IX73, Olympus, Japan).

### Transcriptomics

Following cell cultivation, Trizol was placed on ice, and a microscope (ECLIPSE Ts2, Nikon, Japan) was used to study the morphology of the cells. After washing the cells with PBS and discarding any leftover liquid, 0.5 mL of Trizol was added. To separate the cells, they were gently mixed. Chloroform was added, and the mixture was shaken for 15 s before being left to stand for two minutes at room temperature. After centrifugation at 12,000 rpm for 15 min at 4 °C, the supernatant was collected. After combining this with an equivalent amount of isopropanol, it was allowed to sit at room temperature for ten minutes before being centrifuged for ten minutes. The supernatant was discarded. After adding a volume of 75% ethanol equivalent to Trizol, the sample was centrifuged for five minutes at 4 °C at 7500 rpm. A second wash was carried out after the supernatant was disposed of. After allowing the pellet to air dry in the centrifuge tube, a suitable amount of water treated with DEPC was added to dissolve it. The amount of RNA was measured.

Magnetic beads were used for mRNA enrichment in order to exclude non-target RNAs. Reverse transcription was used to create cDNA from the enriched mRNA, and the library was then built using adaptor ligation and end repair. The target regions were then covered by PCR amplification. Spectrophotometry and electrophoresis were used to evaluate the quality of the library. High-throughput sequencing was performed on the libraries once they were created in accordance with the requirements of the sequencing platform.

Cutadapt was used to trim low-quality sequences, Hisat2 was used to align them to the reference genome, and StringTie was used to reconstruct transcripts and calculate FPKM. Fold change and significance levels were used to determine which genes were differentially expressed. Key biological processes were identified by pathway studies using the Kyoto Encyclopedia of Genes and Genomes (KEGG) and Gene Ontology (GO).

### Co-immunoprecipitation (Co-IP)

Cells (1 × 10^7^ cells/sample) were lysed in ice-cold IP lysis buffer (Beyotime, China) supplemented with protease and phosphatase inhibitors. After incubation on ice for 30 min, cell lysates were centrifuged at 12,000 × *g* for 15 min at 4 °C to remove debris. The supernatant was collected, and protein concentrations were determined using the BCA Protein Assay Kit (Thermo Fisher Scientific, USA). Equal amounts of total protein (500–1000 µg) were incubated with 2–5 µg of the indicated primary antibody or IgG control overnight at 4 °C with gentle rotation. The next day, 40 µL of protein A/G magnetic beads (Thermo Fisher Scientific, USA) were added to each sample and incubated for an additional 2–4 h at 4 °C. Immunocomplexes were then washed three times with cold lysis buffer, resuspended in SDS loading buffer, and boiled for 5 min at 95 °C. Proteins were resolved by SDS-PAGE and analyzed by Western blot using specific antibodies. Input samples (5–10% of total lysate) were used as loading controls.

### Biochemical assays

Whole blood samples (collected from experimental mice) were left undisturbed at room temperature for 30 min to allow for complete clot formation and then centrifuged at 3000 × *g* for 10 min to extract the supernatant. Serum biochemical parameters, including alanine aminotransferase (ALT), aspartate aminotransferase (AST), direct bilirubin (D-BIL), total bilirubin (T-BIL), albumin (ALB), alkaline phosphatase (ALP), total bile acid (TBA), urea (UREA), creatinine (CREA), and uric acid (UA), were measured in a single-reagent format with the R1 reagent. An automated biochemical analyzer (Mindray BS-200, China) was used for the experiments; experimental parameters were pre-programmed to enable automatic sample loading and analysis. For additional processing, the generated data was exported in Excel format.

### Statistical analysis

The statistical analyses were conducted using GraphPad Prism, and the findings are shown as mean ± standard deviation (SD). An independent samples *t*-test was used for comparisons between two groups, and a one-way analysis of variance (ANOVA) was used to evaluate differences between three or more groups, with post hoc testing used as necessary. A *p*-value of less than 0.05 was considered statistically significant. All experiments were carried out in triplicate to guarantee the accuracy and consistency of the results.

## Results

### PFKFB3 is highly expressed in PCa tissues and CRPC cell lines

**Fig. 1:**
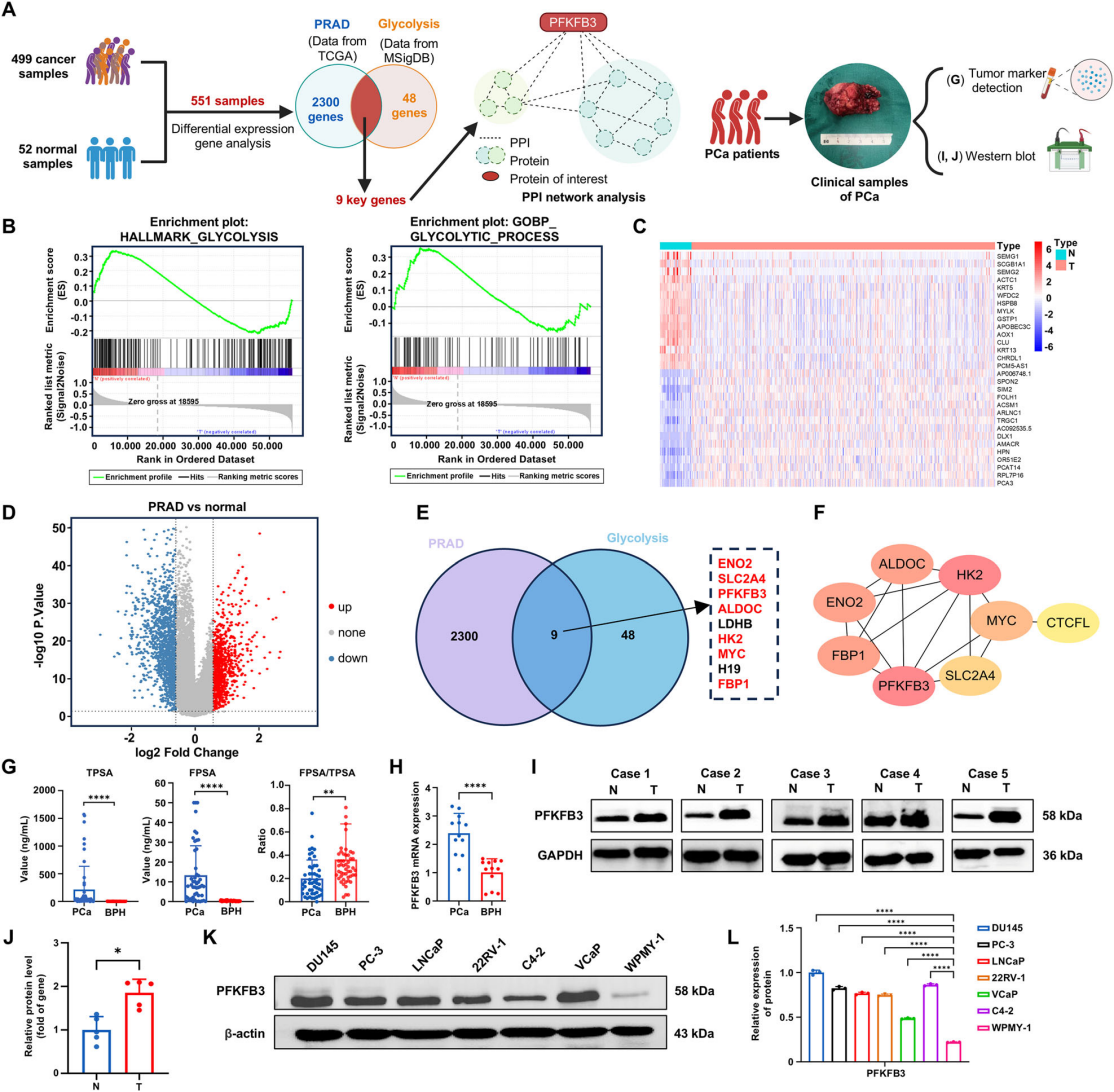
PFKFB3 is highly expressed in PCa tissues and CRPC cell lines. **A** Schematic overview of the clinical bioinformatics analysis and experimental workflow with clinical samples. **B** GSEA results for the glycolysis pathway, showing significant enrichment in PCa. **C** Heatmap of differentially expressed genes in PRAD versus normal samples from the TCGA database, highlighting the most significantly altered genes. **D** Volcano plot of differential gene expression analysis in PRAD compared to normal samples from the TCGA database. Red dots represent upregulated genes, and blue dots represent downregulated genes. **E** Venn diagram showing the intersection between differentially expressed genes in PCa and glycolysis-related genes. **F** PPI based on the STRING database, illustrating the interactions among glycolysis-related proteins. **G** Measurement of PSA levels in the blood of patients with PCa and BPH. **H** qPCR analysis of PFKFB3 mRNA expression levels in prostate tissues from patients with PCa and BPH. **I** Western blot analysis of PFKFB3 protein expression levels in prostate tissues from patients with PCa and BPH. **J** Densitometric quantification of Western blot results shown in (**I**), normalized to GAPDH. **K** Western blot analysis of PFKFB3 protein expression levels in six CRPC cell lines and normal prostate epithelial cells (WPMY-1 is an immortalized human normal prostate stromal cell line, while the others are CRPC cell lines). **L** Densitometric quantification of Western blot results shown in (**K**), normalized to β-actin. The data were presented as the mean ± SD values. ^*^P < 0.05; ^**^P < 0.01, ^****^P < 0.0001 by One-way ANOVA. Related to Fig. S1.

Through systematic analysis of clinical bioinformatics data and experimental validation with clinical samples (Fig. 1A), we established a strong association between abnormal PFKFB3 expression and PCa, highlighting its pivotal role in PCa progression.

The glycolytic pathway in PCa tissues was significantly enriched, according to GSEA (Fig. 1B), indicating that glycolytic metabolism plays a crucial role in PCa development. Differential expression analysis revealed significant gene expression differences between PCa samples from the TCGA database and normal tissues. Heatmap analysis highlighted the most differentially expressed genes (Fig. 1C), providing a foundation for identifying glycolysis-related candidate genes. A volcano plot was used to illustrate the gene distribution, where genes with significant upregulation were represented in red and those with downregulation in blue (Fig. 1D). By intersecting differentially expressed genes with glycolysis-related genes, we discovered a group of genes that were directly associated with glycolysis and markedly elevated in PCa (Fig. 1E). PPI network analysis using the STRING database revealed complex interactions among these genes (Fig. 1F), with PFKFB3 as a central node, underscoring its crucial role in glycolysis and PCa progression. To validate PFKFB3 expression levels in clinical PCa patients, we collected prostate tissue samples from patients with PCa and BPH, using BPH samples as controls due to the small size of surgically excised PCa tissues and the challenge of distinguishing them from adjacent non-cancerous tissues. We first assessed prostate-specific antigen (PSA) levels in patient blood samples and found a strong association between elevated PSA levels and PCa progression (Fig. 1G). qPCR analysis demonstrated a notable elevation in PFKFB3 mRNA expression in PCa tissues compared to BPH tissues (Fig. 1H). H&E staining revealed significant pathological differences in tissue structures between PCa and BPH patients (Fig. S1). These results were consistent with our western blot analysis, confirming high expression of PFKFB3 protein in PCa tissues (Fig. 1I, J).

Six human CRPC cell lines (DU145, PC-3, LNCaP, 22RV-1, C4-2, and VCaP) and normal prostate epithelial cells (WPMY-1) were subjected to western blot analysis to assess the expression of the PFKFB3 protein. The PFKFB3 protein was shown to be much more abundant in CRPC cell lines than in normal cells (Fig. 1K, L), indicating that it plays a critical role in the development of CRPC.

### The regulatory role of PFKFB3 in CRPC cell proliferation and tumor formation

**Fig. 2:**
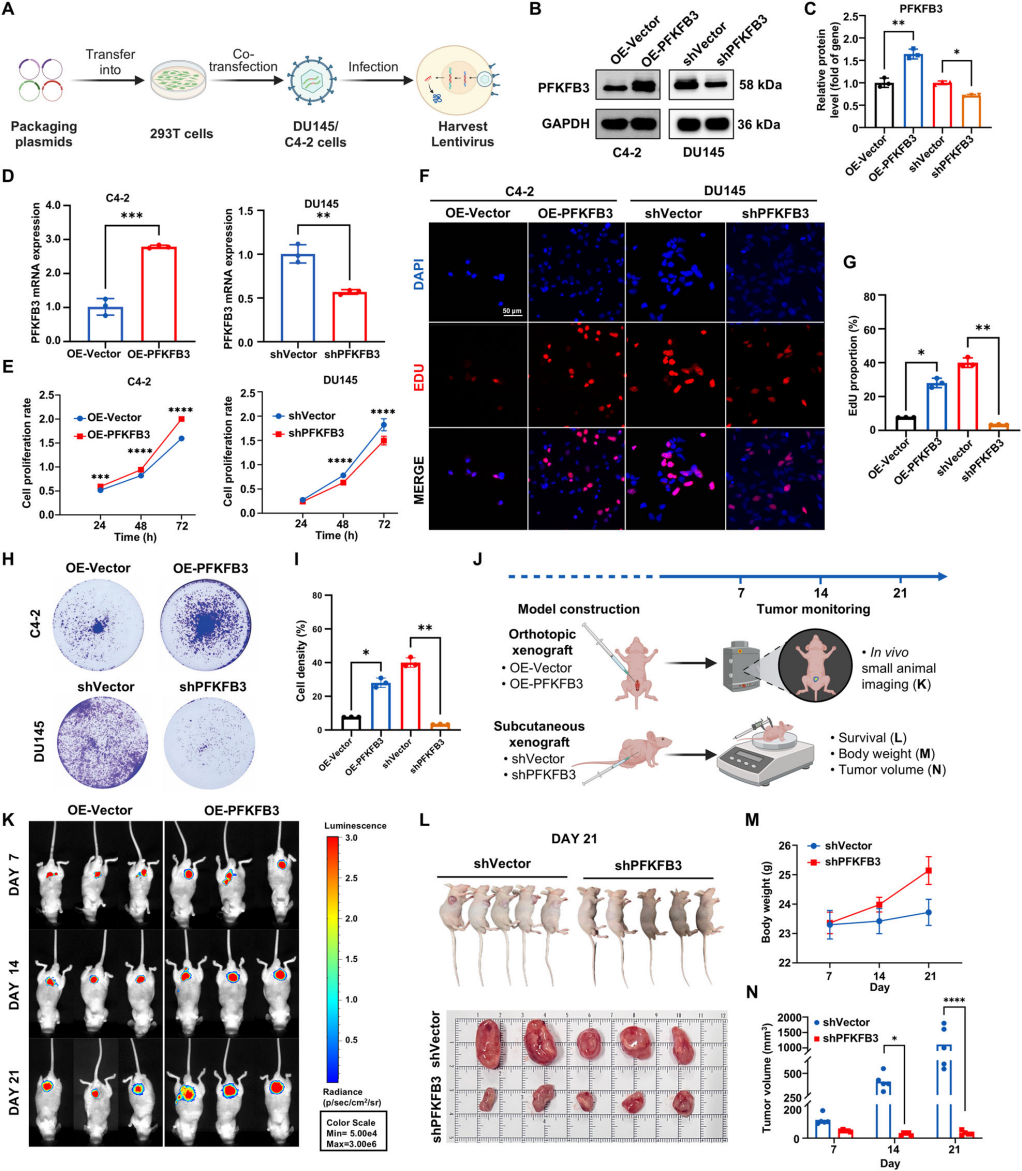
The regulatory role of PFKFB3 in CRPC cell proliferation and tumor formation. **A** Schematic representation of the process for generating shPFKFB3 and OE-PFKFB3 cell lines using lentiviral vectors (shPFKFB3 represents the knockdown group, OE-PFKFB3 indicates the overexpression group, similarly hereafter). **B** Western blot validation of the successful establishment of OE-PFKFB3 in C4-2 cells and shPFKFB3 in DU145 cells. **C** Densitometric quantification of PFKFB3 protein levels from (**B**), normalized to GAPDH. **D** qPCR analysis confirming the construction of OE-PFKFB3 and shPFKFB3 models in C4-2 and DU145 cells, respectively. **E** CCK-8 assay assessing the effect of shPFKFB3 in DU145 and OE-PFKFB3 in C4-2 cells on the cell proliferation rate. **F** EdU assay measuring the impact of shPFKFB3 in DU145 and OE-PFKFB3 in C4-2 cells on cell proliferation. **G** Quantification of EdU-positive cells expressed as a percentage of total cells. **H** Colony formation assay evaluating the effect of shPFKFB3 in DU145 and OE-PFKFB3 in C4-2 cells on colony-forming ability. **I** Quantification of colony numbers from (**H**). **J** Schematic diagram of the tumorigenesis experiment in nude mice with shPFKFB3 in DU145 and OE-PFKFB3 in C4-2 cells, conducted over 21 days. **K** Tumor growth promotion in nude mice after OE-PFKFB3 in C4-2 cells. **L** Tumor growth inhibition in nude mice after shPFKFB3 in DU145 cells. **M** Changes in body weight of nude mice following shPFKFB3. **N** Tumor volume changes in nude mice after shPFKFB3. The data were presented as the mean ± SD values. ^*^P < 0.05; ^**^P < 0.01, ^***^P < 0.001, ^****^P < 0.0001 by One-way ANOVA. Related to Fig. S2.

Fig. 2A illustrates the schematic workflow for constructing OE-PFKFB3 and shPFKFB3 cell models using lentiviral vectors to systematically assess the biological function of PFKFB3. We overexpressed PFKFB3 in C4-2 cells and knocked it down in DU145 cells using these lentiviral vectors. Western blot and qPCR analyses confirmed successful OE-PFKFB3 in C4-2 cells and effective shPFKFB3 in DU145 cells (Fig. 2B–D). We used the CCK-8 assay to evaluate the impact of OE-PFKFB3 in C4-2 cells and shPFKFB3 in DU145 cells on cell proliferation. While OE-PFKFB3 considerably boosted proliferation in C4-2 cells, shPFKFB3 significantly decreased proliferation in DU145 cells (Fig. 2E). Furthermore, EdU assays, which assess DNA synthesis as a proliferation marker, yielded consistent results, further confirming the role of PFKFB3 in promoting cell proliferation (Fig. 2F, G). Immunofluorescence staining revealed a substantial decrease in PFKFB3 levels in DU145 cells, while C4-2 cells exhibited a notable elevation (Fig. S2), further supporting the pivotal role of PFKFB3 in modulating cell proliferation. To investigate the role of PFKFB3 in tumor formation, we conducted colony formation assays. DU145 and C4-2 cells underwent shPFKFB3 and OE-PFKFB3, respectively, and were cultured in six-well plates for two weeks. We used crystal violet staining to count and analyze the resulting colonies (Fig. 2H, I). shPFKFB3 in DU145 cells significantly inhibited colony formation, while OE-PFKFB3 in C4-2 cells enhanced it, confirming PFKFB3’s importance in CRPC cell growth and tumorigenesis.

In vivo, we performed tumorigenesis experiments in nude mice to evaluate the role of PFKFB3 in tumor growth. We injected DU145 cells (shPFKFB3 group) subcutaneously into the flanks and C4-2 cells (OE-PFKFB3 group) orthotopically into the prostate. We monitored tumor growth over 21 days (Fig. 2J). The results indicated that OE-PFKFB3 in C4-2 cells significantly accelerated tumor proliferation in vivo (Fig. 2K), whereas shPFKFB3 in DU145 cells effectively suppressed tumor growth (Fig. 2L). Notably, shPFKFB3 did not cause significant weight loss in mice, and tumor volume was substantially reduced (Fig. 2M, N), further supporting PFKFB3’s potential as a therapeutic target in CRPC.

### Impact of PFKFB3 on cell cycle progression, apoptosis, and organelle integrity in CRPC

**Fig. 3:**
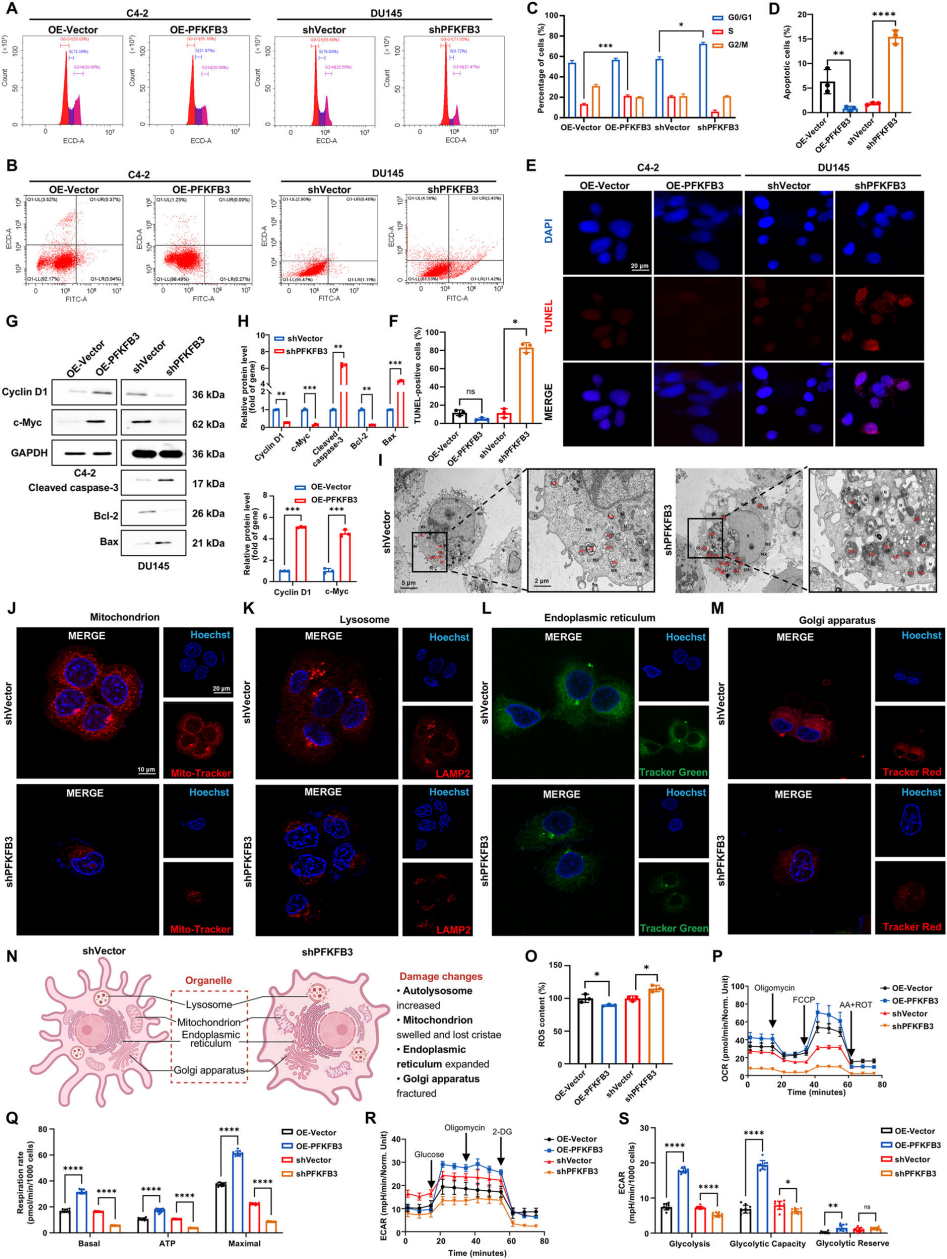
Impact of PFKFB3 on cell cycle progression, apoptosis, and organelle integrity in CRPC. **A** Schematic representation of cell cycle changes following shPFKFB3 in DU145 cells and OE-PFKFB3 in C4-2 cells. **B** Flow cytometry analysis showing the impact of shPFKFB3 in DU145 cells and OE-PFKFB3 in C4-2 cells on apoptosis. **C** Quantification of the cell cycle data. **D** Quantification of apoptosis, expressed as the percentage of apoptotic cells, based on flow cytometry data. **E** TUNEL assay results illustrate DNA fragmentation in DU145 cells with shPFKFB3 and C4-2 cells with OE-PFKFB3. **F** Quantification of DNA fragmentation in DU145 and C4-2 cells, based on TUNEL assay. **G** Western blot analysis of cell cycle and apoptosis markers in DU145 and C4-2 cells following shPFKFB3 and OE-PFKFB3. **H** Densitometric quantification of cell cycle and apoptosis markers from (**G**), normalized to GAPDH. **I** Electron microscopy showing organelle damage in DU145 cells with shPFKFB3. pseudopodia (PS), nucleus (N), nucleolus (Nu), mitochondria (M), dilated rough endoplasmic reticulum (RER) with ribosomes attached to the membrane, golgi apparatus (GO), lipid droplets (LD), autophagolysosomes (ASS). **J** Laser confocal microscopy showing mitochondrial damage in DU145 cells following shPFKFB3. **K** Laser confocal microscopy showing lysosomal fragmentation in DU145 cells with shPFKFB3. **L** Laser confocal microscopy showing endoplasmic reticulum dilation in DU145 cells after shPFKFB3. **M** Laser confocal microscopy showing Golgi apparatus fragmentation in DU145 cells with shPFKFB3. **N** Schematic illustration summarizing organelle damage induced by shPFKFB3 in DU145 cells. **O** ROS analysis in DU145 and C4-2 cells showing the effects of shPFKFB3 and OE-PFKFB3 on cellular oxidative stress. **P** Seahorse OCR analysis in DU145 and C4-2 cells showing the effects of shPFKFB3 and OE-PFKFB3 on mitochondrial respiration. **Q** Quantification of OCR data from (**P**), normalized to 5000 cells. **R** Seahorse ECAR analysis in DU145 and C4-2 cells illustrating the effects of shPFKFB3 and OE-PFKFB3 on glycolytic activity. **S** Quantification of ECAR data from (**R**), normalized to 5000 cells. The data were presented as the mean ± SD values. ^*^P < 0.05; ^**^P < 0.01, ^***^P < 0.001, ^****^P < 0.0001, ns no significant difference by One-way ANOVA. Related to Figs. S3 and S4.

By assessing its effects on DU145 cells with shPFKFB3 and C4-2 cells with OE-PFKFB3, we investigated the role of PFKFB3 on cell cycle progression and apoptosis in CRPC cells. Flow cytometric analysis revealed significant alterations in cell cycle distribution. shPFKFB3 in DU145 cells resulted in a marked cell cycle arrest, evidenced by a substantial increase in the proportion of cells in the G0/G1 phase (Fig. 3A). This implies that in order to affect cellular proliferation, PFKFB3 may directly alter signaling pathways linked to the cell cycle. When PFKFB3 was overexpressed in C4-2 cells, the percentage of cells in the S phase significantly increased (Fig. 3C), further reinforcing its involvement in facilitating cell cycle progression and promoting tumor cell proliferation.

Next, we assessed the impact of PFKFB3 on apoptosis using flow cytometry and TUNEL assays. shPFKFB3 in DU145 cells significantly increased the proportion of apoptotic cells, whereas its OE-PFKFB3 in C4-2 cells markedly reduced apoptosis (Fig. 3B, D). These results were corroborated by the TUNEL assay, which showed a significant increase in TUNEL-positive cells in DU145 cells upon shPFKFB3, indicating elevated DNA fragmentation and apoptosis (Fig. 3E, F). Western blot analysis confirmed these findings, revealing that PFKFB3 regulates key proteins involved in cell cycle progression and apoptosis. Specifically, shPFKFB3 resulted in a decrease in Cyclin D1 and c-Myc expression, both of which are critical for promoting cell cycle progression. In contrast, OE-PFKFB3 in C4-2 cells led to increased levels of these proteins, further supporting its role in driving cell cycle progression. Moreover, we detected alterations in apoptosis-related markers, where silencing PFKFB3 led to a notable increase in the pro-apoptotic protein Bax and a reduction in the anti-apoptotic protein Bcl-2, thereby enhancing cell death. The activation of caspase-3, a key executioner of apoptosis, was also observed following shPFKFB3, confirming the induction of apoptosis (Fig. 3G, H). These findings highlight the role of PFKFB3 in regulating the cell cycle and apoptosis by influencing the expression of key proteins, including Cyclin D1, c-Myc, Bax, Bcl-2, and caspase-3.

To further investigate the underlying mechanisms, we performed electron microscopy to examine the structural integrity of cellular organelles following shPFKFB3. Our analysis revealed extensive organelle damage, which suggests impaired mitochondrial function and compromised energy production (Fig. 3I). These results suggest that PFKFB3 is critical for maintaining the structural integrity of cellular organelles. Further analysis using laser confocal microscopy provided visual evidence of morphological alterations in multiple organelles following shPFKFB3, including the mitochondria (Fig. 3J), lysosomes (Fig. 3K), endoplasmic reticulum (Fig. 3L), and the Golgi apparatus (Fig. 3M). Mitochondrial fragmentation suggests potential impairment in energy metabolism. Lysosomal swelling and reduced granularity may reflect altered autophagic processes, while changes in endoplasmic reticulum and Golgi morphology are indicative of stress responses or disruption in protein processing. Additionally, the accumulation of lipid droplets (Fig. S3) was observed in shPFKFB3 cells, further indicating an alteration in cellular metabolism and lipid homeostasis. These results collectively demonstrate the critical function of PFKFB3 in maintaining cellular homeostasis and organelle function, underscoring its importance in the metabolic control of CRPC cells.

Fig. 3N provides a schematic illustration of the organelle damage induced by shPFKFB3, summarizing the observed structural impairments in mitochondria, lysosomes, endoplasmic reticulum, and the Golgi apparatus. This diagram further emphasizes the critical role of PFKFB3 in maintaining cellular integrity and metabolic function.

Additionally, our analysis of ROS revealed that PFKFB3 regulates cellular oxidative stress responses, suggesting that PFKFB3 may be involved in modulating cellular stress and metabolic homeostasis. Further supporting this, ROS fluorescence staining and co-localization analysis demonstrated an increased ROS accumulation in shPFKFB3 cells, highlighting a disruption in oxidative stress management (Figs. 3O and S4). This co-localization of ROS with organelles, such as mitochondria, further underscores the role of PFKFB3 in maintaining cellular redox balance and its potential involvement in the regulation of cellular stress pathways. To further elucidate the metabolic alterations associated with PFKFB3, Seahorse assays were performed. OCR analysis (Fig. 3P, Q) showed that shPFKFB3 significantly impaired mitochondrial respiration, while ECAR measurements (Fig. 3R, S) indicated reduced glycolytic activity. These findings reinforce the conclusion that PFKFB3 plays a crucial role in coordinating oxidative stress responses and cellular energy metabolism, potentially through regulation of both mitochondrial function and glycolytic flux.

### Impact of PFKFB3 on CRPC cell migration and metastasis

**Fig. 4:**
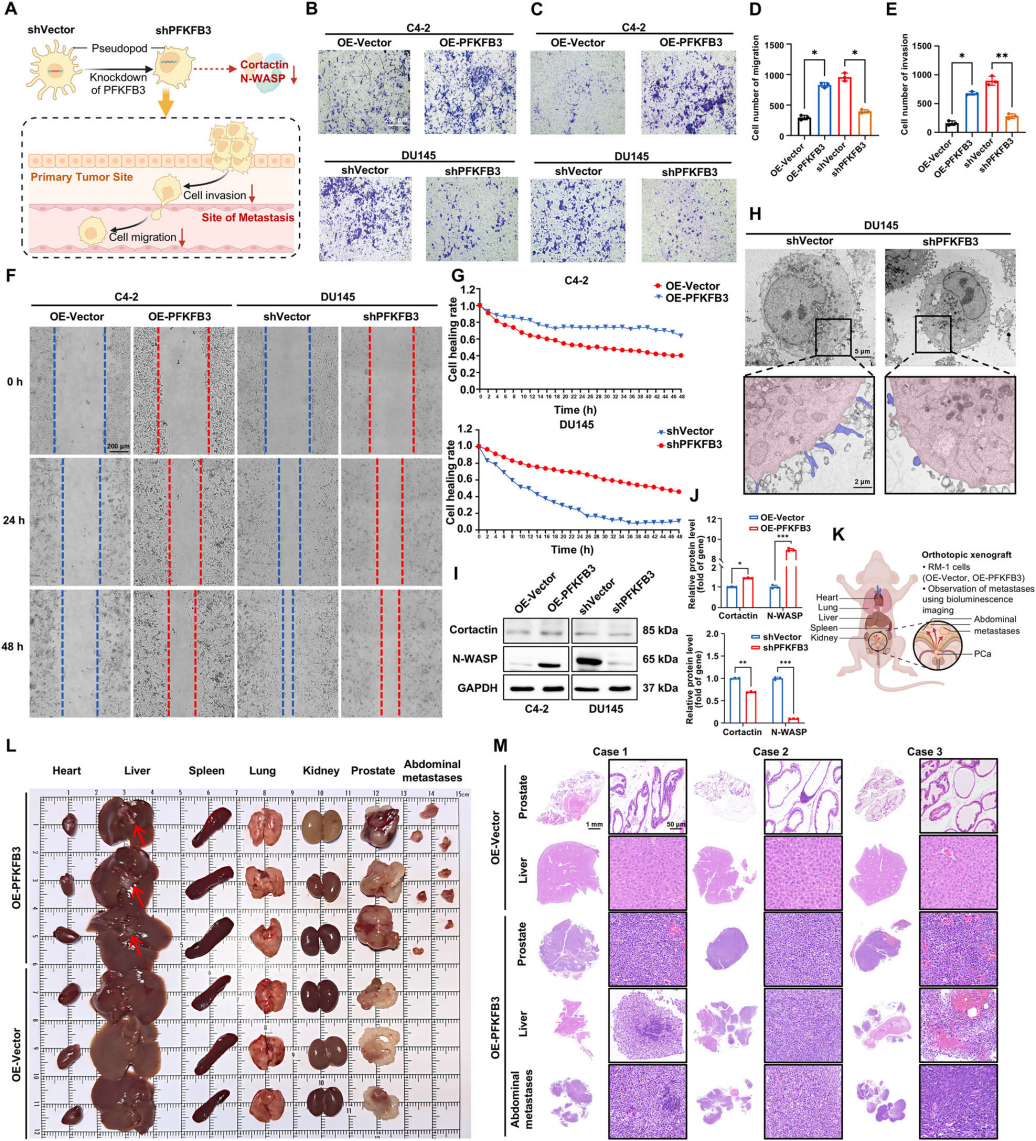
Impact of PFKFB3 on CRPC cell migration and metastasis. **A** Schematic representation of the cellular morphological and behavioral changes following shPFKFB3 in DU145 cells, highlighting reduced migratory and invasive capacities. **B** Transwell migration assay showing reduced migration in DU145 cells with shPFKFB3 and increased migration in C4-2 cells with OE-PFKFB3. **C** Transwell invasion assay demonstrating inhibited invasion in DU145 cells with shPFKFB3 and enhanced invasion in C4-2 cells with OE-PFKFB3. **D** Quantification of migrated cells from (**B**), based on Transwell assay results. **E** Quantification of invaded cells from (**C**), based on Transwell assay results. **F** Wound healing assay showing reduced migration in DU145 cells with shPFKFB3 and enhanced migration in C4-2 cells with OE-PFKFB3. **G** Quantification of cell migration in the wound healing assay from (**F**). **H** Electron microscopy analysis showing structural changes in DU145 cells after shPFKFB3, indicating alterations in cellular morphology. **I** Western blot analysis of Cortactin and N-WASP expression in DU145 cells with shPFKFB3, revealing decreased levels of these key cytoskeletal regulators. **J** Densitometric quantification of Cortactin and N-WASP levels from (**I**), normalized to GAPDH. **K** Schematic representation of organ-specific metastasis in nude mice following orthotopic injection of RM-1 cells with OE-PFKFB3. **L** Gross images of the heart, liver, spleen, lung, kidney, prostate tissues, and metastatic lesions dissected from mice three weeks after establishing an orthotopic PCa model using RM-1 cells overexpressing PFKFB3. The metastatic lesions were observed in the abdominal cavity of the mice. **M** H&E staining of the prostate, liver, and metastatic lesions in nude mice after orthotopic injection of RM-1 cells overexpressing PFKFB3. The data were presented as the mean ± SD values. ^*^P < 0.05; ^**^P < 0.01, ^***^P < 0.001 by One-way ANOVA. Related to Fig. S5.

Fig. 4A presents a schematic representation of the cellular morphological and behavioral changes following shPFKFB3 in DU145 cells, highlighting a significant reduction in both migratory and invasive capacities. To further investigate these effects, we performed transwell assays to assess the migration and invasion abilities of DU145 cells with shPFKFB3 and C4-2 cells with OE-PFKFB3. The results revealed that shPFKFB3 in DU145 cells notably inhibited migration and invasion, while OE-PFKFB3 in C4-2 cells enhanced these abilities (Fig. 4B–E), suggesting a potential role for PFKFB3 in promoting CRPC metastasis.

The idea that PFKFB3 is a crucial regulator of cellular architecture and motility was further supported by electron microscopy analysis, which revealed notable changes in the morphology of DU145 cells following shPFKFB3 (Fig. 4H). We used western blot analysis to measure the expression levels of two proteins linked to pseudopodia, Cortactin and N-WASP, in order to look into the molecular processes causing these changes. Cortactin is essential for pseudopodia formation and cell migration, while N-WASP plays a critical role in cytoskeletal remodeling and pseudopodia dynamics [23, 24]. Our findings indicate that silencing PFKFB3 led to a marked reduction in the expression of Cortactin and N-WASP in DU145 cells (Fig. 4I, J), further supporting the role of PFKFB3 in regulating cell migration and invasion via these critical cytoskeletal regulators.

In order to corroborate these results, we used a real-time wound healing experiment to track the migration patterns of C4-2 cells with OE-PFKFB3 and DU145 cells with shPFKFB3 (Fig. 4F, G). Consistent with the earlier results, shPFKFB3 impaired the ability of DU145 cells to close the wound, whereas OE-PFKFB3 in C4-2 cells accelerated wound closure, highlighting the enhanced migratory potential associated with PFKFB3. This functional assay further corroborates our protein-level observations and strengthens the evidence linking PFKFB3 to cell migration.

In vivo, the metastatic potential of OE-PFKFB3 in RM-1 cells was assessed using an orthotopic PCa model in nude mice. As illustrated in the schematic diagram (Fig. 4K), control or OE-PFKFB3 in RM-1 cells were orthotopically injected into the prostates of male nude mice to mimic the tumor microenvironment and monitor spontaneous metastasis. Remarkably, mice in the OE-PFKFB3 group developed visible liver metastases (Fig. 4L), indicating that PFKFB3 significantly enhances the metastatic capability of CRPC cells in vivo. In addition to liver involvement, metastatic tumor nodules were also observed within the abdominal cavity, further supporting the widespread dissemination of tumor cells. This observation was confirmed by histopathological analysis (Fig. 4M), which revealed the presence of metastatic lesions in both the liver and intraperitoneal tissues, thereby validating the occurrence of distant metastases. These results strongly suggest that OE-PFKFB3 facilitates the systemic spread of CRPC cells, including to the liver and abdominal cavity—key steps in cancer progression.

Taken together, our findings demonstrate that PFKFB3 plays a critical role not only in regulating cell proliferation but also in enhancing cell motility, invasion, and in vivo metastatic potential. These results identify PFKFB3 as a crucial driver of CRPC progression and provide new insights into its role in promoting tumor infiltration and liver metastasis.

### Investigating the regulatory mechanisms of PFKFB3 in CRPC cells

**Fig. 5:**
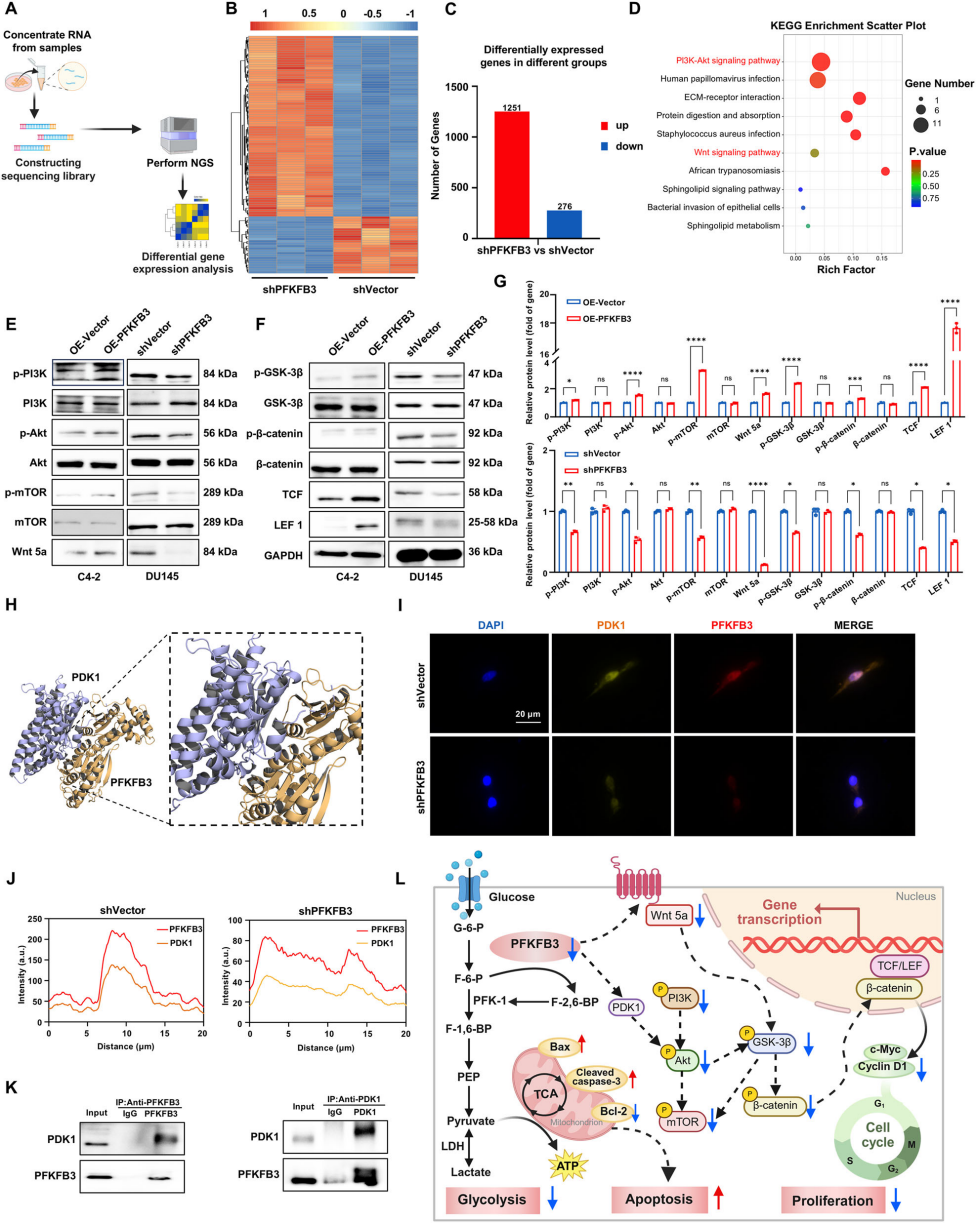
Investigating the regulatory mechanisms of PFKFB3 in CRPC cells. **A** Schematic diagram of the shPFKFB3 experiment in DU145 cells for transcriptomic analysis. **B** Heatmap of differentially expressed genes in DU145 cells following shPFKFB3, based on transcriptomic analysis. **C** List of differentially expressed genes identified in the transcriptomic analysis. **D** KEGG pathway enrichment analysis of the differentially expressed genes from the transcriptomic data. **E**, **F** Western blot validation of changes in protein expression levels corresponding to the transcriptomic findings. **G** Densitometric quantification of protein expression changes shown in (**E**, **F**). **H** Molecular docking experiments to identify direct interaction targets of PFKFB3. **I** Immunofluorescence co-localization of PFKFB3 and PDK1 in DU145 cells following shPFKFB3. **J** Quantitative analysis of PFKFB3 and PDK1 co-localization in DU145 cells shown in (**I**). **K** Co-IP analysis of the interaction between PFKFB3 and PDK1 in DU145 cells following shPFKFB3. **L** Schematic representation of the molecular mechanism of PFKFB3 in CRPC cells. The data were presented as the mean ± SD values. ^*^P < 0.05; ^**^P < 0.01, ^***^P < 0.001, ^****^P < 0.0001, ns no significant difference by One-way ANOVA. Related to Figs. S6 and S7.

We further investigated the regulatory mechanisms of PFKFB3 in CRPC cells. A shPFKFB3 model was established in DU145 cells, serving as a basis for subsequent analyses of gene expression changes. According to transcriptomic analysis, shPFKFB3 markedly changed the gene expression profile, leading to the upregulation of 1251 genes and the downregulation of 276 genes (Figs. 5A–C and S6). Additional examination of these genes with variable expression revealed a high correlation with the development and course of CRPC. Silencing PFKFB3 changed important intracellular signaling networks, including the PI3K/Akt and Wnt/β-catenin pathways, which are essential for tumor cell migration, survival, and proliferation, according to KEGG pathway enrichment analysis (Fig. 5D).

The PI3K/Akt pathway is among the most frequently activated signaling pathways in human tumors and regulates various cellular metabolic processes to meet the metabolic demands of tumor cells [25, 26]. This pathway directly regulates glucose metabolism through the phosphorylation of metabolic enzymes, promoting glycolysis [27, 28]. Additionally, the PI3K/Akt pathway is essential for apoptosis regulation, as it reduces the survival of tumor cells by activating pro-apoptotic factors such as Bax and caspase-3. Additionally, the Wnt/β-catenin pathway plays a major role in the development of tumors, especially when it comes to controlling the cell cycle [29]. By activating downstream β-catenin, this pathway controls the production of genes linked to the cell cycle, including Cyclin D1, which promotes the development of the cell cycle and aids in the growth of tumor cells [30, 31].

We used western blot analysis to verify alterations in the protein expression of the pertinent differentially expressed genes in order to investigate the regulatory mechanisms of PFKFB3 on the PI3K/Akt and Wnt/β-catenin pathways in more detail (Fig. 5E–G). The findings indicated a reduction of PI3K signaling activity as shPFKFB3 significantly decreased the levels of PI3K and its phosphorylated form, p-PI3K. This change was accompanied by reduced levels of Akt and its phosphorylated form, p-Akt, further confirming pathway inhibition. Furthermore, the downregulation of mTOR and its phosphorylated form, p-mTOR, indicated diminished proliferative signaling, affecting the stability of downstream targets, GSK-3β and β-catenin. Reduced expression of proliferation-associated proteins, including as c-Myc and Cyclin D1, resulted from the inhibition of β-catenin’s association with TCF/LEF1. GSK-3β, a shared regulatory component in both pathways, indicates that PFKFB3 regulates both the Wnt/β-catenin and PI3K/Akt pathways.

In our study, shPFKFB3 resulted in increased Bax expression and decreased Bcl-2 expression, suggesting that PFKFB3 suppression promotes apoptotic signaling. Akt phosphorylates and inhibits Bax, reducing its pro-apoptotic effects. Additionally, Akt phosphorylates Bcl-2, which enhances its anti-apoptotic function. Consequently, decreased activity of the PI3K/Akt signaling pathway, along with the upregulation of Bax and downregulation of Bcl-2, predisposes cells to apoptosis. These results suggest that PFKFB3 promotes the proliferation of CRPC cells and inhibits apoptosis by regulating the PI3K/Akt/mTOR and Wnt/β-catenin pathways.

Given that Akt is a central component of the PI3K/Akt signaling pathway and that PFKFB3 has been implicated in regulating Akt phosphorylation, we investigated the potential interaction between PFKFB3 and key upstream regulators of Akt activation, including the rapamycin complex 2 (mTORC2) and 3-phosphoinositide-dependent protein kinase 1 (PDK1) [32]. Molecular docking experiments revealed interactions between PFKFB3 and both mTORC2 and PDK1, highlighting potential mechanisms through which PFKFB3 regulates Akt phosphorylation (Figs. 5H and S7). mTORC2 is a critical component of the mTOR signaling pathway, regulating cell growth, metabolism, and survival [33, 34]. Specifically, PFKFB3 may promote the phosphorylation and activation of Akt, thereby facilitating the activation of downstream signaling pathways that drive cell proliferation and survival. PDK1 plays a critical role in this process by directly phosphorylating Akt at residues such as Thr308, which is essential for maintaining metabolic homeostasis and supporting cell survival in response to extracellular stimuli such as nutrients, oxygen, and growth factors [35, 36]. The potential interaction between PFKFB3 and PDK1 implies that PFKFB3 may contribute not only to metabolic reprogramming but also to the enhancement of Akt signaling activity, thereby promoting tumor cell proliferation and survival. These findings provide new insights into the role of PFKFB3 in cancer cell growth and anti-apoptotic mechanisms. To further explore the molecular basis of this regulatory mechanism, we Co-IP experiments to investigate the potential binding partners of PFKFB3. The results revealed that PFKFB3 can interact with PDK1 (Fig. 5K), suggesting a direct or indirect physical association between the two proteins. Although the precise nature of this interaction requires further validation, this result supports the hypothesis that PFKFB3 may influence PI3K/Akt pathway activity via interaction with PDK1. In addition, immunofluorescence staining showed co-localization of PFKFB3 and PDK1 within the cytoplasm (Fig. 5I, J), providing further spatial evidence of their potential interaction. These findings collectively suggest that PFKFB3 may modulate Akt phosphorylation by interacting with PDK1, thereby contributing to the activation of the PI3K/Akt/mTOR signaling pathway and promoting cancer cell survival and progression.

Figure 5L summarizes the regulatory mechanisms of PFKFB3 in CRPC cells, highlighting its critical role in promoting cell proliferation. These findings challenge the traditional view of PFKFB3 as solely a metabolic regulator and underscore its critical role in signal transduction, reinforcing the potential of PFKFB3 as a therapeutic target in CRPC. These findings highlight the distinct function of PFKFB3 in CRPC and offer a fresh theoretical framework for creating focused treatment plans to combat the illness.

### Evaluation of the clinical significance of PFKFB3 as a potential therapeutic target in CRPC treatment

**Fig. 6:**
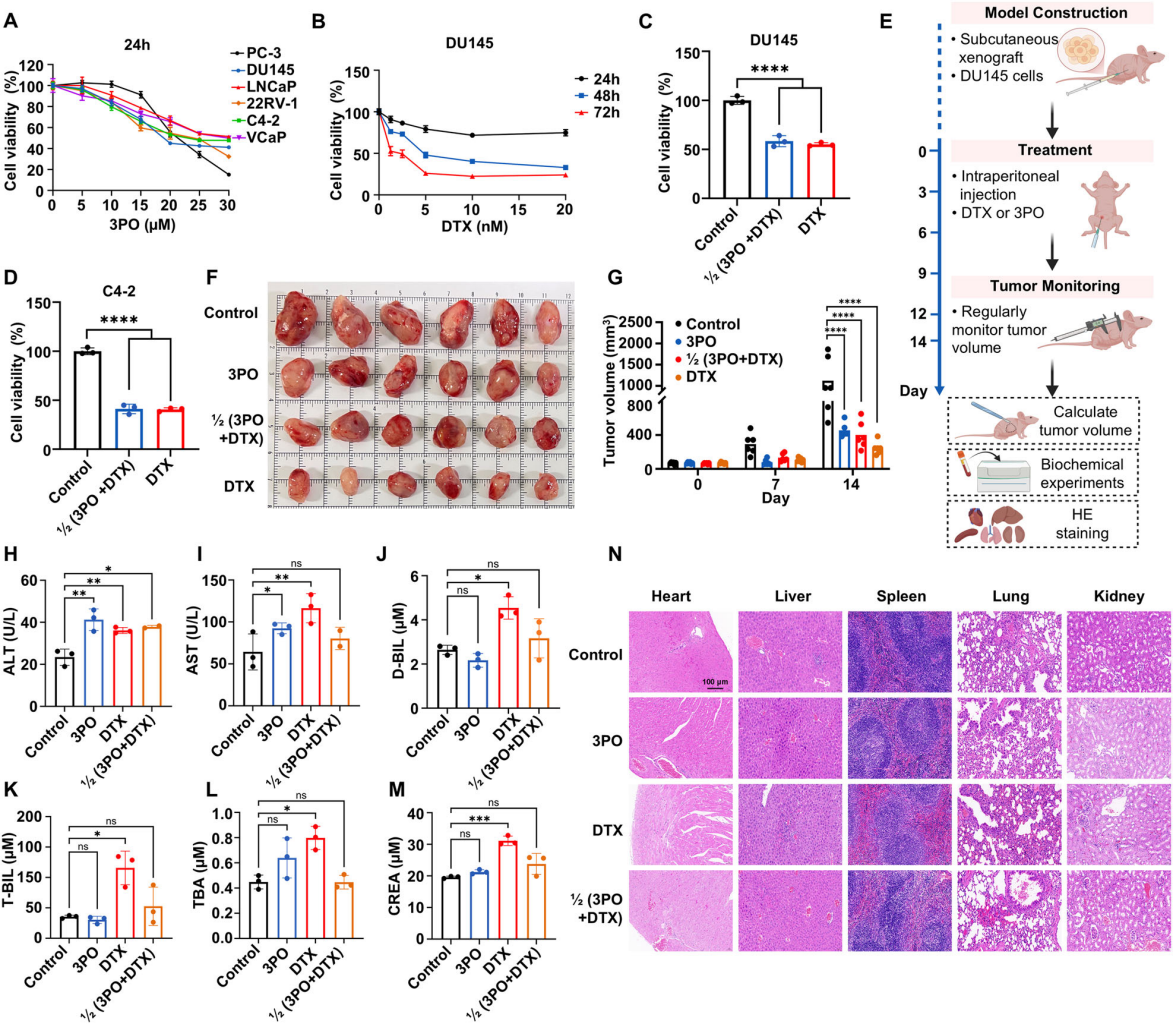
Evaluation of the clinical significance of PFKFB3 as a potential therapeutic target in CRPC treatment. **A** The effect of the PFKFB3 inhibitor 3PO on cell proliferation was assessed in six CRPC cell lines (PC-3, DU145, LNCaP, 22RV-1, C4-2, and VCaP) after 24 h of treatment. The IC_50_ values (μM) at 24 h were as follows: PC-3 (21.59), DU145 (22.63), LNCaP (28.87), 22RV-1 (21.65), C4-2 (25.86), and VCaP (29.56). **B** Changes in the proliferation rate of DU145 cells treated with various concentrations of DTX (nM) for 24, 48, and 72 h. The calculated IC_50_ values for DU145 cells were 3.291, 3.293, and 1.519 nM, respectively. **C**, **D** Synergistic effects of combined treatment with 3PO and DTX on the proliferation of DU145 (**C**) and C4-2 (**D**) CRPC cells. Cells were treated with either agent alone at their respective IC_50_ concentrations or in combination at 0.5 × IC_50_ of each drug for 48 h. The IC_50_ values for 3PO were 17.04 μM for DU145 and 11.09 μM for C4-2, and for DTX were 3.293 nM for DU145 and 0.9386 nM for C4-2. Accordingly, the concentrations used for the combination treatment were 8.52 μM 3PO and 1.6465 nM DTX for DU145 cells, and 5.545 μM 3PO and 0.4693 nM DTX for C4-2 cells. **E** Schematic diagram of the in vivo experimental workflow in nude mice, illustrating efficacy validation of 3PO, DTX, and their combination. **F** Subcutaneous tumor tissues excised from nude mice after 14 days, grouped into control, 3PO, DTX, and combined treatment groups. **G** Tumor volume changes in nude mice after treatment administration. **H–L** Biochemical analyses of liver function parameters (ALT, AST, D-BIL, T-BIL, TBA) across different groups. **M** Biochemical analyses of kidney function parameters (CREA) across different groups. **N** Histopathological analysis of heart, liver, spleen, lung, and kidney tissues in each treatment group using H&E staining. The data were presented as the mean ± SD values. ^*^P < 0.05; ^**^P < 0.01, ^***^P < 0.001, ^****^P < 0.0001, ns no significant difference by One-way ANOVA. Related to Fig. S8.

In order to assess the effectiveness and safety of this unique approach to treating CRPC, this research thoroughly examined the therapeutic potential of the PFKFB3 inhibitor 3PO, both as a stand-alone treatment and in conjunction with the first-line chemotherapeutic drug DTX. We conducted in vitro experiments on six CRPC cell lines treated with 3PO for 24, 48, and 72 h. The crucial function of PFKFB3 in CRPC cell development is shown by (Figs. 6A and S8A, B), which show that 3PO dramatically decreased cell proliferation at all time periods. The effectiveness of both medicines was further supported by DTX’s time-dependent inhibitory impact on DU145 and C4-2 cell growth (Figs. 6B and S8C). However, DTX is associated with significant toxicity, limiting its clinical application [37].

The co-treatment of 3PO and DTX demonstrated notable synergistic effects in both DU145 and C4-2 cell lines (Fig. 6C, D). This combination therapy enhanced the sensitivity of cancer cells to DTX, inhibiting tumor growth, while also potentially reducing DTX toxicity and improving treatment safety.

We validated our in vitro findings in vivo using a mouse xenograft model (Fig. 6E). Monitoring tumor volume in the control, 3PO, DTX, and combination therapy groups (Fig. 6F, G) revealed that combination therapy significantly inhibited tumor growth. We performed biochemical analyses of liver function indicators in each group (Figs. 6H–L and S8D, E), measuring ALT, AST, D-BIL, T-BIL, TBA, ALB, and ALP. The results showed that in the DTX monotherapy group, several liver function indicators were significantly elevated, indicating hepatic damage. Specifically, elevated levels of ALT, AST, D-BIL, T-BIL, and TBA were observed, suggesting liver injury induced by DTX. Similarly, analysis of kidney function indicators among different groups (Figs. 6M and S8F, G) revealed increased CREA levels in the DTX group, indicative of renal dysfunction. These findings confirm that DTX causes both liver and kidney damage. However, the combination of 3PO and DTX significantly mitigated these toxic effects. Importantly, achieving the same therapeutic effect with half the dosage of DTX in the combination treatment effectively reduces the overall toxicity associated with DTX, highlighting the potential of 3PO to enhance treatment safety. Figure 6N shows the histological examination of the heart, liver, spleen, lungs, and kidneys. In the DTX monotherapy group, significant histopathological changes were observed, including signs of tissue damage such as hepatocellular necrosis, myocardial degeneration, and mild renal tubular injury, all of which are commonly associated with the high toxicity of DTX. In contrast, the combination treatment of 3PO and DTX significantly reduced these pathological changes, with minimal signs of organ damage in the heart, liver, spleen, lungs, and kidneys, suggesting a protective effect provided by the PFKFB3 inhibitor. It should be noted that although DTX is efficient in suppressing tumors, it is very toxic and can have negative effects on key organs. On the other hand, PFKFB3 inhibitors like 3PO exhibit considerably lower toxicity, as demonstrated by the preserved organ architecture and reduced pathological changes. The combination of 3PO and DTX not only alleviates the toxic side effects typically seen with DTX monotherapy, but it also maintains tumor growth inhibition efficacy comparable to DTX alone. This synergistic effect suggests that 3PO can enhance the therapeutic index of DTX by improving its safety profile while maintaining, or even enhancing, its anticancer efficacy. This finding is crucial for developing combination strategies that maximize therapeutic benefits while minimizing adverse effects, thus improving the overall treatment safety and quality of life for patients.

In conclusion, DTX and the PFKFB3 inhibitor 3PO together demonstrated exceptional safety by lowering DTX toxicity while successfully suppressing CRPC cell growth. According to our results, PFKFB3 appears to be a viable therapeutic target for CRPC, offering a solid basis for research on precision-targeted treatments and enhancing CRPC management. Targeted therapy against PFKFB3 holds promise for improving the prognosis of CRPC patients, enhancing treatment efficacy while minimizing adverse effects, and providing new insights for personalized medicine.

## Discussion

In the last ten years, CRPC has emerged as a major challenge in PCa therapy [38, 39]. PCa is a malignancy highly reliant on androgen signaling, and while ADT is initially effective in most patients, many ultimately progress to a castration-resistant state, known as CRPC. At this stage, standard endocrine therapies lose efficacy, leading to substantially increased risks of recurrence and metastasis [40, 41]. Given the limited and often ineffective treatment options for CRPC, identifying novel molecular targets is critical for advancing our understanding of its pathophysiology and developing more effective therapies. Recently, metabolic reprogramming has emerged as a key feature of cancer progression, with particular relevance in androgen-dependent cancers like PCa [42, 43]. The development and progression of CRPC are closely linked to alterations in several metabolic pathways, particularly glycolysis [44]. CRPC cells enhance glycolytic activity to meet the heightened energy demands of rapid proliferation. These metabolic alterations not only strengthen tumor cell proliferation and survival but also drive treatment resistance and invasiveness, thereby accelerating disease progression and recurrence [45–47]. Elucidating the metabolic regulatory mechanisms in CRPC is thus crucial for understanding its biological characteristics and for advancing novel therapeutic approaches. In this context, our study is the first to reveal the key role of the glycolytic regulator PFKFB3 in CRPC, expanding our understanding of its potential function in PCa regulation. Among glycolytic regulators, PFKFB3 has been identified as a key player in CRPC, yet its role relative to other PFK2 isoforms remains underexplored. Previous studies have reported the involvement of PFKFB2 and PFKFB4 in PCa metabolism, suggesting that multiple PFK2 isoforms contribute to glycolytic regulation. Additionally, these PFK2 isoforms likely regulate overlapping downstream signaling pathways [13, 14]. PFKFB3-mediated glycolytic activation has been linked to the PI3K/Akt and Wnt/β-catenin signaling pathways, both of which are critical for PCa progression. Notably, PFKFB4 has also been reported to modulate the Akt and MYC signaling cascades, suggesting a potential convergence of metabolic and oncogenic networks. Whether PFKFB2 and PFKFB4 regulate key cellular processes such as filopodia and lamellipodia formation, as PFKFB3 does, remains an open question. Given the importance of actin cytoskeleton remodeling in cancer invasion and metastasis, future studies should investigate the role of these isoforms in cytoskeletal dynamics. Targeting glycolytic regulators like PFKFB3 represents a promising strategy for metabolic intervention in CRPC. 3PO, a small-molecule inhibitor of PFKFB3, has demonstrated antitumor effects by suppressing glycolysis and impairing cancer cell proliferation. However, its specificity and pharmacokinetic properties remain limitations for clinical application. More recently, next-generation PFKFB3 inhibitors with improved selectivity and bioavailability have been developed, some of which are currently undergoing preclinical evaluation. These compounds hold potential for combination therapies aimed at overcoming metabolic plasticity in CRPC.

Thus, our study provides novel insights into PFKFB3 as a key glycolytic regulator in CRPC, emphasizing its distinct role among PFK2 isoforms. Further investigations into the functional interactions of these isoforms, their downstream signaling overlap, and the translational potential of PFKFB3 inhibitors will be essential for advancing metabolic-targeted therapies in CRPC.

Previous research highlights the importance of glycolysis in tumor growth; in this study, GSEA enrichment analysis and PPI network construction confirmed the central role of PFKFB3 in regulating glycolysis-related gene expression in PCa. Through in vivo and in vitro experiments, we assessed the role of PFKFB3 in CRPC cell proliferation and migration. OE-PFKFB3 significantly promoted cell proliferation, while its knockdown markedly inhibited both proliferation and migration, confirming its pro-carcinogenic role in CRPC. Furthermore, our findings demonstrate that PFKFB3 influences organelle morphology, underscoring its role in regulating cell apoptosis and energy metabolism. Transcriptomic analysis showed significant gene expression changes upon shPFKFB3, with pathway enrichment analysis highlighting its involvement in various metabolic and signaling pathways. Innovatively, this study is the first to reveal PFKFB3’s dual regulatory effects on the PI3K/Akt and Wnt/β-catenin pathways, and it shows that PFKFB3 promotes tumor cell proliferation and cell cycle progression synergistically by inhibiting GSK-3β and reducing Cyclin D1 and c-Myc expression. This intersectional pathway regulation illustrates PFKFB3’s bridging role between metabolism and signaling. Additionally, molecular docking experiments unveiled for the first time a direct interaction between PFKFB3 and PDK1, suggesting that by enhancing Akt phosphorylation activity, PFKFB3 may promote tumor cell proliferation and resistance to apoptosis. This finding challenges the conventional view of PFKFB3 as a singular metabolic enzyme, revealing its dual function in CRPC cell metabolism and apoptosis regulation. Ultimately, this study provides a comprehensive elucidation of PFKFB3’s complex roles in glycolysis, cell proliferation, apoptosis, and signaling pathways, challenging traditional functional perceptions and offering novel theoretical support for targeting PFKFB3 in CRPC therapy. This finding highlights a promising target for precision therapy in CRPC, offering considerable clinical translational potential.

This study further elucidates the synergistic efficacy and toxicity-reducing effects of the PFKFB3 inhibitor 3PO combined with DTX in the treatment of CRPC, offering an innovative complement to existing therapeutic approaches and a promising avenue for future personalized treatment strategies. In particular, the combination therapy strategy, which reduces the DTX dosage by half, not only alleviates the adverse effects associated with high-dose chemotherapy but also addresses the challenge of drug resistance that arises from prolonged treatment. Elevated PFKFB3 expression may be a promising clinical biomarker for determining CRPC malignancy and chemotherapy resistance, allowing for the early detection of high-risk patients and the creation of individualized and precise treatment plans. Moreover, the unique mechanism of PFKFB3 as a metabolic target highlights its potential as a pivotal entry point in the field of cancer metabolism, thereby advancing the development of anti-cancer metabolic therapies. As metabolic targeting therapy gains traction in cancer treatment, PFKFB3 not only expands the therapeutic target landscape in CRPC but also provides new exploratory directions within the realm of metabolic regulation.

## Conclusions

This study systematically investigated the critical role of the glycolytic regulator PFKFB3 in CRPC. Through GSEA enrichment analysis and PPI network construction, PFKFB3 was identified as a central regulator of glycolysis-related gene expression in PCa. Both in vitro and in vivo experiments further confirmed that OE-PFKFB3 significantly promoted CRPC cell proliferation, whereas its knockdown markedly inhibited cell proliferation and migration while affecting organelle morphology. These findings underscore PFKFB3’s pivotal role in regulating apoptosis and energy metabolism. Transcriptomic analysis revealed significant changes in gene expression following shPFKFB3, with enrichment in multiple metabolic and signaling pathways. Notably, this study is the first to uncover PFKFB3’s dual regulatory effects on the PI3K/Akt and Wnt/β-catenin signaling pathways. PFKFB3 was shown to inhibit tumor cell proliferation and cell cycle progression by regulating the phosphorylation of GSK-3β, thereby reducing the expression of Cyclin D1 and c-Myc. Molecular docking experiments demonstrated a direct interaction between PFKFB3 and Akt phosphorylation-regulated proteins PDK1, with shPFKFB3 suppressing Akt phosphorylation and promoting apoptosis in CRPC cells.

Additionally, the combination of the PFKFB3 inhibitor 3PO and DTX exhibited remarkable synergistic efficacy and reduced toxicity, providing an innovative strategy for CRPC treatment. Combining DTX with 3PO to reduce its dosage while maintaining effective tumor suppression minimized the adverse effects of prolonged high-dose chemotherapy and alleviated drug resistance. This study not only provides theoretical support for targeting PFKFB3 as a novel therapeutic approach for CRPC but also highlights its potential as a clinical biomarker for identifying high-risk patients and guiding personalized treatment strategies.

## Supplementary information

Supplementary Materials

Supplementary figureS1

Supplementary figureS2

Supplementary figureS3

Supplementary figureS4

Supplementary figureS5

Supplementary figureS6

Supplementary figureS7

Supplementary figureS8

## Data Availability

All data generated or analyzed during this study are included in this published article and its Supplementary Information or from the corresponding author upon reasonable request.
