# Supplementary material for: PFKFB3 as a multifaceted driver and therapeutic target in castration-resistant prostate cancer
Source: Cell Death Dis. 2025 Oct 24;16(1):760. doi: 10.1038/s41419-025-08089-8 (PMC12552466; doi:10.1038/s41419-025-08089-8)
Supplement: Supplementary file 1 — Supplementary Materials [file 41419_2025_8089_MOESM1_ESM.docx]

**Supplementary Materials for**

PFKFB3 as a multifaceted driver and therapeutic target in castration-resistant prostate cancer

Lin Chen ^a,b†^, Yu-Xin Xu ^a,b†^, Ying-Ying Ren ^a,b†^, Zhi-Da Wang ^c^, Xue-Man Dong ^a,b^, Yi-Min Chen ^a,b^, Pu Wu ^a,b^, Tong Wu ^a,b^, Fei Xiang ^c^, Tian Xie ^a,b*^, Qi Zhang ^c*^, Jian-Liang Zhou ^a,b*^

Correspondence to: cpuzhou@163.com, cpuzhou@hznu.edu.cn; clinic@126.com; xbs@hznu.edu.cn

**1. Supplementary figure legends**

Fig. S1. PFKFB3 is highly expressed in PCa tissues and CRPC cell lines (related to Fig. 1).

Fig. S2. The regulatory role of PFKFB3 in CRPC cell proliferation and tumor formation (related to Fig. 2).

Fig. S3. Impact of PFKFB3 on cell cycle progression, apoptosis, and organelle integrity in CRPC (related to Fig. 3).

Fig. S4. Impact of PFKFB3 on cell cycle progression, apoptosis, and organelle integrity in CRPC (related to Fig. 3).

Fig. S5. Impact of PFKFB3 on CRPC cell migration and metastasis (related to Fig. 4).

Fig. S6. Investigating the regulatory mechanisms of PFKFB3 in CRPC cells (related to Fig. 5).

Fig. S7. Investigating the regulatory mechanisms of PFKFB3 in CRPC cells (related to Fig. 5).

Fig. S8. Evaluation of the clinical significance of PFKFB3 as a potential therapeutic target in CRPC treatment (related to Fig. 6).

**2. Supplementary tables**

Table S1. List of antibodies used in this study.

Table S2. Nucleotide sequences of qPCR primers.

Table S3. Nucleotide sequences of shRNA.

Table S4. Information on patients with PCa and BPH

**Fig. S1. PFKFB3 is highly expressed in PCa tissues and CRPC cell lines (related to Fig. 1).** HE staining showing pathological changes in prostate tissues from patients with PCa and BPH.

**Fig. S2. The regulatory role of PFKFB3 in CRPC cell proliferation and tumor formation (related to Fig. 2).** Immunofluorescence assay showing the localization of PFKFB3 protein expression in DU145 cells with shPFKFB3 and C4-2 cells with OE-PFKFB3.

**Fig. S3. Impact of PFKFB3 on cell cycle progression, apoptosis, and organelle integrity in CRPC (related to Fig. 3).** Changes in lipid droplets observed by laser confocal microscopy in DU145 cells after PFKFB3 knockdown.

**Fig. S4. Impact of PFKFB3 on cell cycle progression, apoptosis, and organelle integrity in CRPC (related to Fig. 3).** Changes in reactive oxygen species (ROS) observed by laser confocal microscopy in DU145 cells with PFKFB3 knockdown and C4-2 cells with PFKFB3 overexpression.

**Fig. S5. Impact of PFKFB3 on CRPC cell migration and metastasis (related to Fig. 4).** Cell migration analysis via wound healing assay after PFKFB3 knockdown and overexpression in DU145 and C4-2 cells, respectively.

**Fig. S6. Investigating the regulatory mechanisms of PFKFB3 in CRPC cells (related to Fig. 5).** **(A)** Heatmap of differentially expressed genes in DU145 cells after PFKFB3 knockdown, based on transcriptomic analysis. **(B)** Pearson correlation coefficients between samples in transcriptomic analysis following PFKFB3 knockdown in DU145 cells. **(C)** KEGG pathway enrichment analysis of differentially expressed genes identified in the transcriptomic study.

**Fig. S7. Investigating the regulatory mechanisms of PFKFB3 in CRPC cells (related to Fig. 5).** **(A)** Molecular docking experiments to investigate the direct interaction between PFKFB3 and PDK1. **(B)** Molecular docking experiments to investigate the direct interaction between PFKFB3 and mTORC2.

**Fig. S8. Evaluation of the clinical significance of PFKFB3 as a potential therapeutic target in CRPC treatment (related to Fig. 6).** **(A-B)** The effect of the PFKFB3 inhibitor 3PO on cell proliferation was assessed in six CRPC cell lines (PC-3, DU145, LNCaP, 22RV-1, C4-2, and VCaP) after 48 and 72 hours of treatment. The IC_50_ values (μM) at each time point were as follows: at 48 h, PC-3 (20.84), DU145 (17.04), LNCaP (21.59), 22RV-1 (15.07), C4-2 (11.09), and VCaP (17.47); and at 72 h, PC-3 (17.79), DU145 (17.59), LNCaP (21.59), 22RV-1 (19.47), C4-2 (8.97), and VCaP (14.66). **(C)** Changes in the proliferation rate of C4-2 cells treated with various concentrations of DTX (nM) for 24, 48, and 72 hours. The calculated IC_50_ values for C4-2 cells at 24, 48, and 72 hours were 0.9899, 0.9386, and 0.9358 nM, respectively. **(D-E)** Biochemical analyses of liver function parameters (ALB, ALP) across different groups. **(F-G)** Biochemical analyses of kidney function parameters (UREA, UA) across different groups.

**Table S1. List of antibodies used in this study.**

| **Antibody** | **Source** | **Cat no.** |
| --- | --- | --- |
| Anti-PFKFB3 antibody [EPR12594] | Abcam | ab181861 |
| Anti-GAPDH antibody [EPR16891] – Loading Control | Abcam | ab181602 |
| Anti-beta Catenin (phospho S675) antibody [EPR28410-45] | Abcam | ab314450 |
| Anti-Bcl-2 antibody [E17] | Abcam | ab182858 |
| Anti-Bax antibody [E63] | Abcam | ab182733 |
| Anti-Cleaved Caspase-3 antibody [EPR21032] | Abcam | ab214430 |
| Anti-Caspase-9 antibody [EPR18108] | Abcam | ab185719 |
| Anti-GSK3 beta antibody [3D10] | Abcam | ab93926 |
| Anti-Cyclin D1 antibody [SP4] | Abcam | ab16663 |
| Anti-Cortactin antibody [EP1922Y] | Abcam | ab81208 |
| Anti-N WASP antibody [EPR6959] | Abcam | ab126626 |
| Anti-mTOR antibody [EPR390(N)] | Abcam | ab134903 |
| Anti-mTOR (phospho S2448) antibody [EPR426(2)] | Abcam | ab109268 |
| Anti-PDK1 antibody [EPR19571] | Abcam | ab202468 |
| Beta Actin Monoclonal Antibody | Proteintech | 66009-1-lg |
| c-Myc (D84C12) Rabbit mAb | Cell Signaling Technology | 5605 |
| LEF1 (C12A5) Rabbit mAb | Cell Signaling Technology | 2230 |
| TCF4/TCF7L2 (C48H11) Rabbit mAb | Cell Signaling Technology | 2569 |
| β-Catenin (6B3) Rabbit mAb | Cell Signaling Technology | 9582 |
| Cleaved Caspase-9 (Asp330) (D2D4) Rabbit mAb | Cell Signaling Technology | 7237 |
| Phospho-GSK-3β (Ser9) (5B3) Rabbit mAb | Cell Signaling Technology | 9323 |
| Phospho-PI3 Kinase p85 (Tyr458)/p55 (Tyr199) Antibody | Cell Signaling Technology | 4228 |
| PI3 Kinase p85 (19H8) Rabbit mAb | Cell Signaling Technology | 4257 |
| Akt (pan) (11E7) Rabbit mAb | Cell Signaling Technology | 4685 |
| Phospho-Akt (Thr308) Antibody | MCE | HY-P80789 |
| Wnt5a Antibody | MCE | HY-P80933 |
| Goat anti-Rabbit IgG, Fluor 488 | ThermoFisher | A32731TR |
| Goat anti-Mouse lgG, Fluor 568 | ThermoFisher | A-11031 |

**Table S2. Nucleotide sequences of qPCR primers.**

| ***Names*** | ***Species*** | ***Sequence (5’ to 3’)*** |
| --- | --- | --- |
| *PFKFB3-F* | *Homo sapiens* | GGTCGGAAGAGTGGACTTTG |
| *PFKFB3-R* | *Homo sapiens* | CAGGGTTTGAGGCAATGAG |
| *β-actin-F* | *Homo sapiens* | AAGGTGACAGCAGTCGGTT |
| *β-actin-R* | *Homo sapiens* | TGTGTGGACTTGGGAGAGG |

**Table S3. Nucleotide sequences of shRNA.**

| ***Names*** | ***Species*** | ***Sequence (5’ to 3’)*** |
| --- | --- | --- |
| *PFKFB3* shRNA | *Homo sapiens* | CGGGTGCATGATTGTGCTTAA |
| NC shRNA | *Homo sapiens* | GGACTATCATATGCTTACCG |

**Table S4. Information on patients with PCa and BPH.**

| **PCa, patient number** | **Age** | **Gleason score** | **WHO/ISUP grading group** | **Gleason4 grade** | **Proportion of total PSA (μg/L)** | **Free PSA (μg/L)** |
| --- | --- | --- | --- | --- | --- | --- |
| 90192342 | 65 | 7 | 2/5 group | About 20% | 0.472 | 6.64 |
| 91922287 | 75 | 9 | 5/5 group | >50% | 19.93 | 2.42 |
| 91904774 | 62 | 7 | 2/5 group | About 5% | 0.014 | ＜0.010 |
| 91946498 | 71 | 7 | 3/5 group | About 80% | 12.56 | 0.867 |
| 91893787 | 73 | 9 | 5/5 group | >50% | 30.48 | 4.54 |
| 91949891 | 74 | 8 | 4/5 group | 0% | 40.09 | 2.31 |
| 91867714 | 74 | 7 | 2/5 group | About 30% | 11.49 | 1.03 |
| 91939657 | 64 | 7 | 3/5 group | About 85% | 10.34 | 1.8 |
| 91955243 | 68 | 7 | 3/5 group | >50% | 10.37 | 1.06 |
| 91923156 | 75 | 7 | 2/5 group | About 20% | 5.42 | 0.708 |
| 90367413 | 76 | 7 | 3/5 group | About 80% | 6.19 | 0.84 |

| **BPH, patient number** | **Age** | **The prostate tissue was resected by transurethral resection** | **Proportion of total PSA (μg/L)** | **Free PSA (μg/L)** |
| --- | --- | --- | --- | --- |
| 15082095 | 54 | 3*1.5*1.5 cm | 0.582 | 0.151 |
| 12586846 | 56 | 2*2*0.5 cm | 3.69 | 0.617 |
| 91873107 | 71 | 10*9*4 cm | 14.81 | 4.12 |
| 15035274 | 77 | 2*2*1 cm | 0.504 | 0.28 |
| 91924737 | 76 | 8*4*2.7 cm | 17.69 | 5.34 |
| 91848560 | 70 | 1.5*1*0.6 cm | 1.05 | 0.157 |
| 91882258 | 68 | 5*3*0.8 cm | 0.679 | 0.174 |
| 91882930 | 77 | 9*8*2 cm | 6.23 | 1.8 |
| 91940925 | 57 | 2*1*0.4 cm | 0.765 | 0.203 |
