# Supplementary figures and images for: PFKFB3 as a multifaceted driver and therapeutic target in castration-resistant prostate cancer
Source: Cell Death Dis. 2025 Oct 24;16(1):760. doi: 10.1038/s41419-025-08089-8 (PMC12552466; doi:10.1038/s41419-025-08089-8)

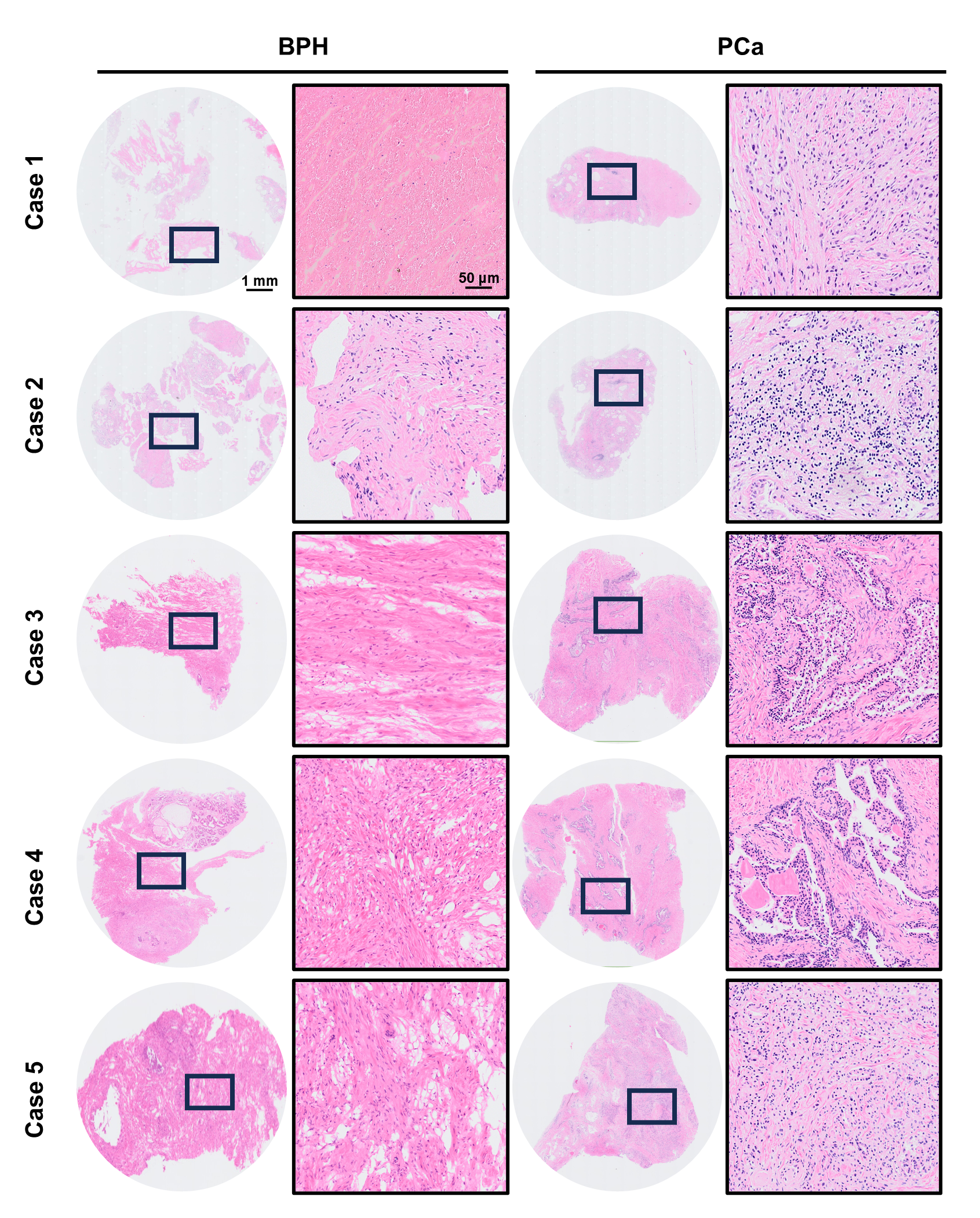

Supplement: Supplementary file 2 — Supplementary figureS1 [file 41419_2025_8089_MOESM2_ESM.png]

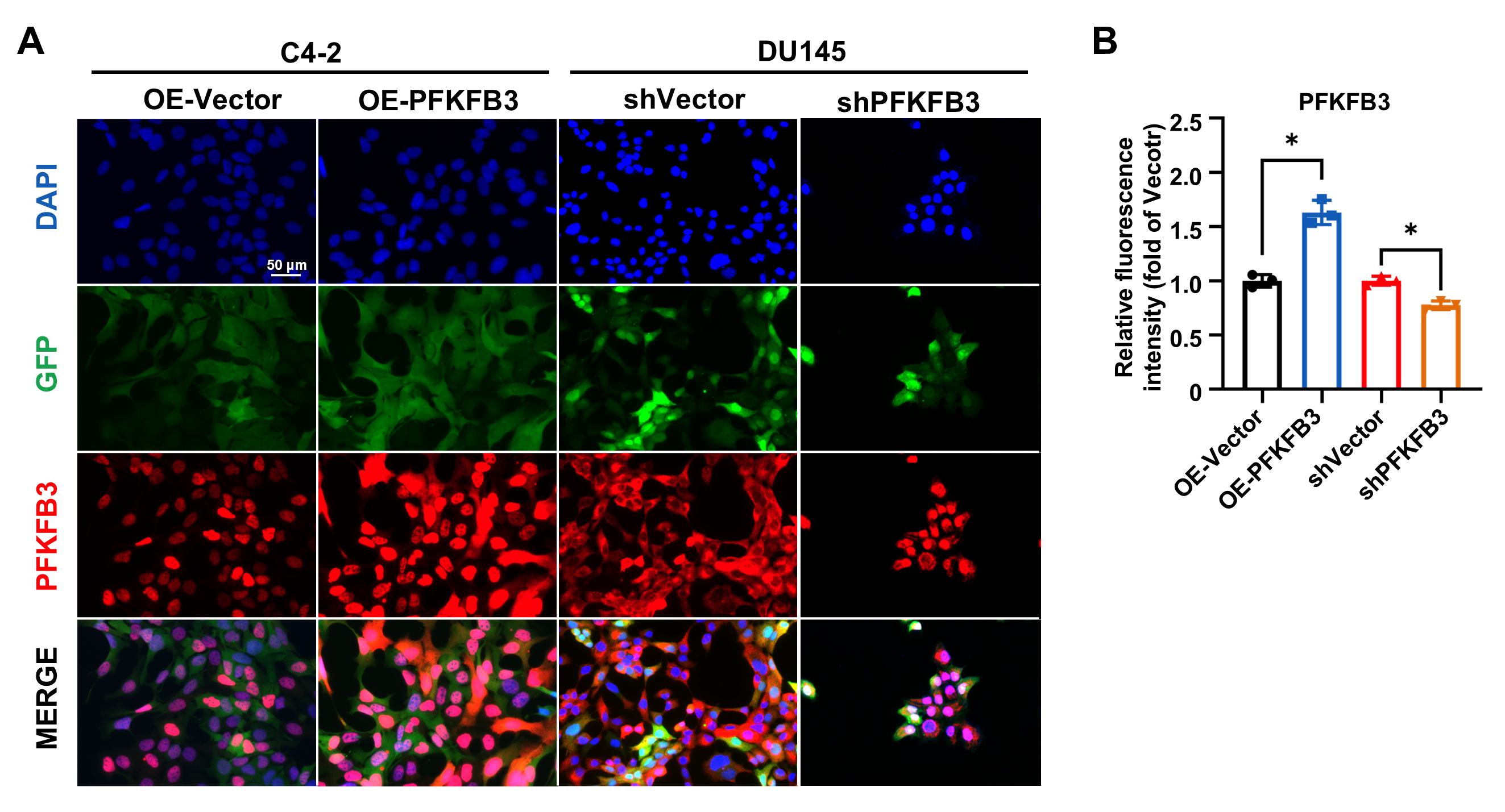

Supplement: Supplementary file 3 — Supplementary figureS2 [file 41419_2025_8089_MOESM3_ESM.png]

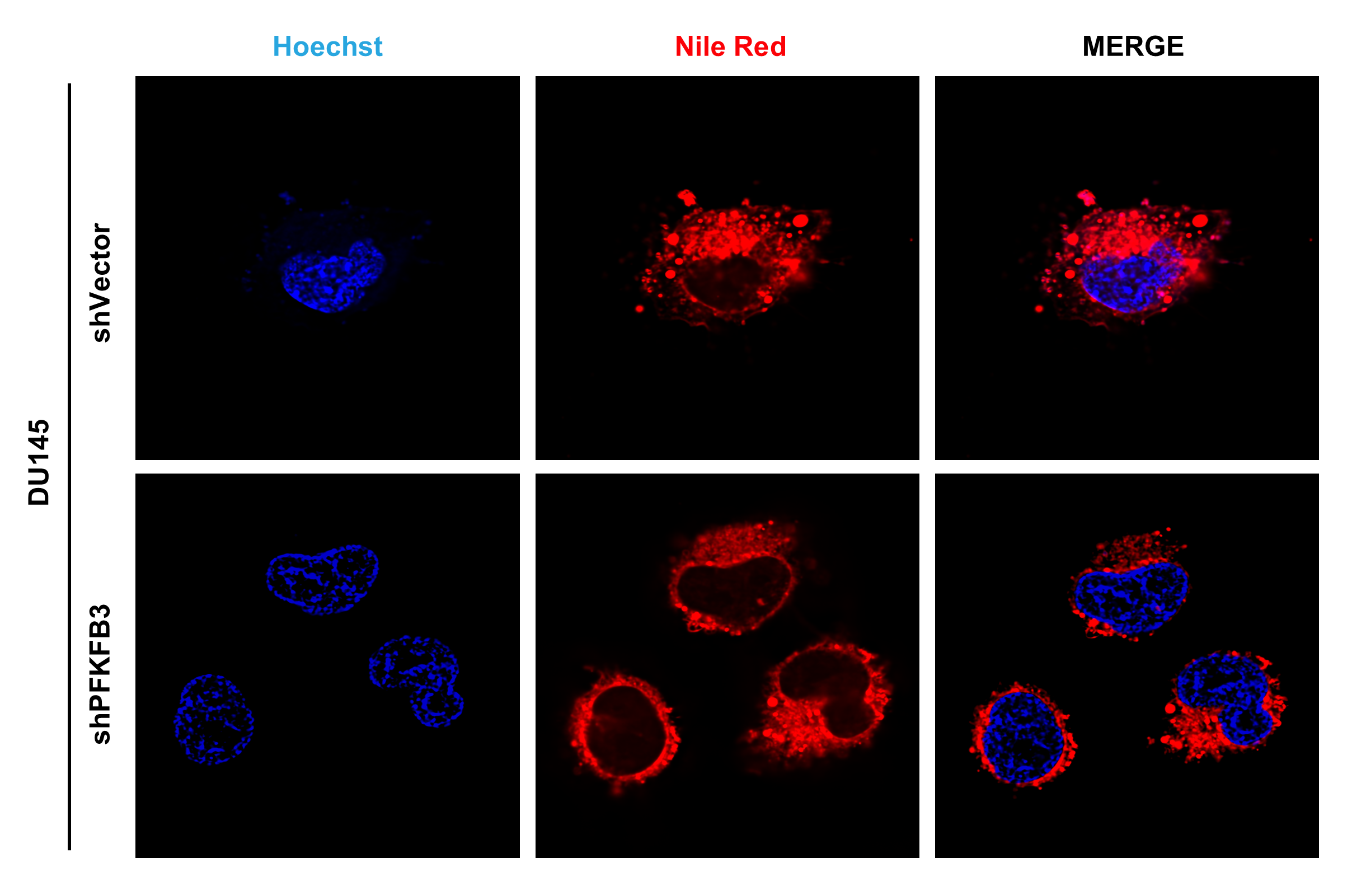

Supplement: Supplementary file 4 — Supplementary figureS3 [file 41419_2025_8089_MOESM4_ESM.png]

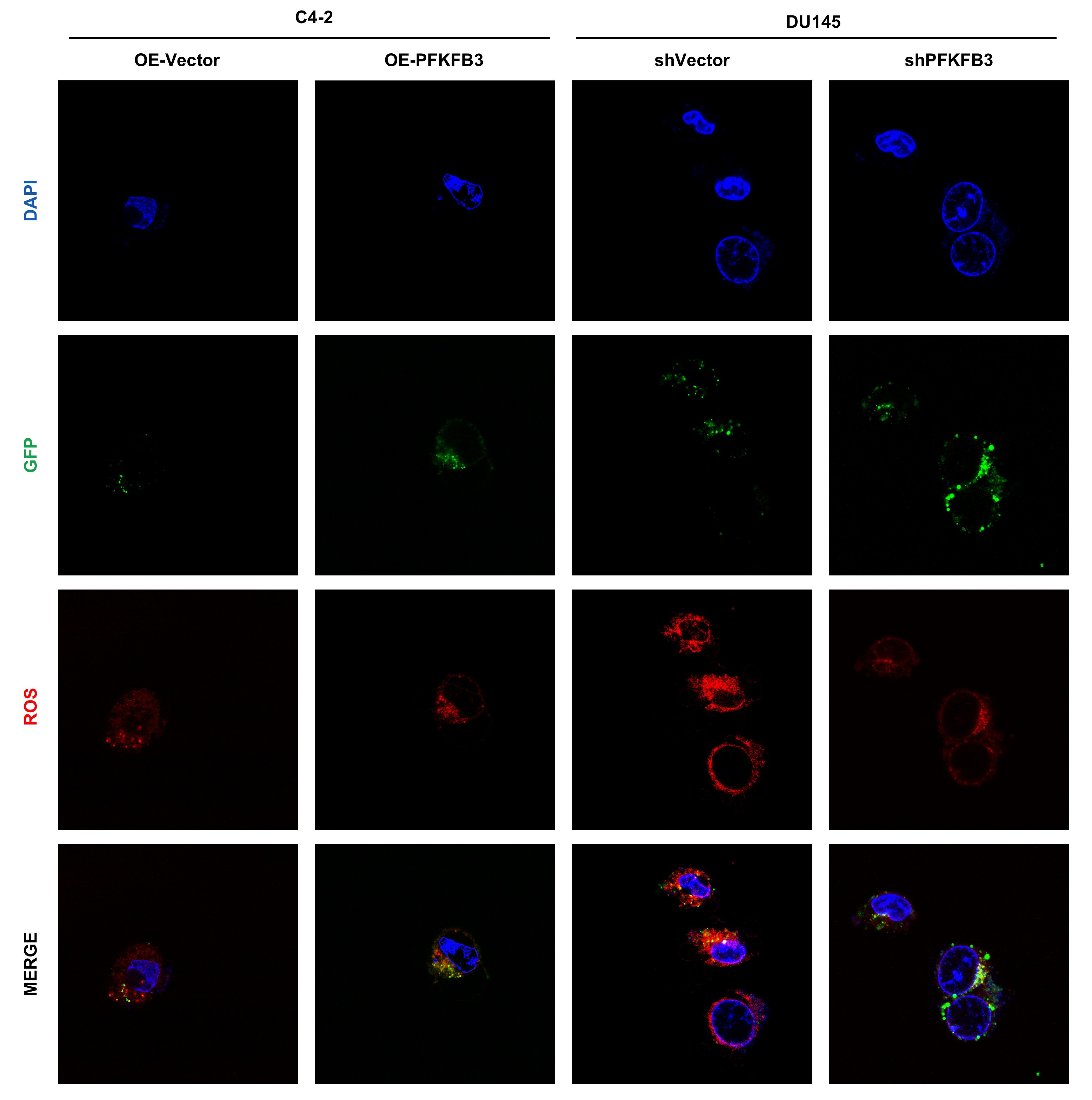

Supplement: Supplementary file 5 — Supplementary figureS4 [file 41419_2025_8089_MOESM5_ESM.png]

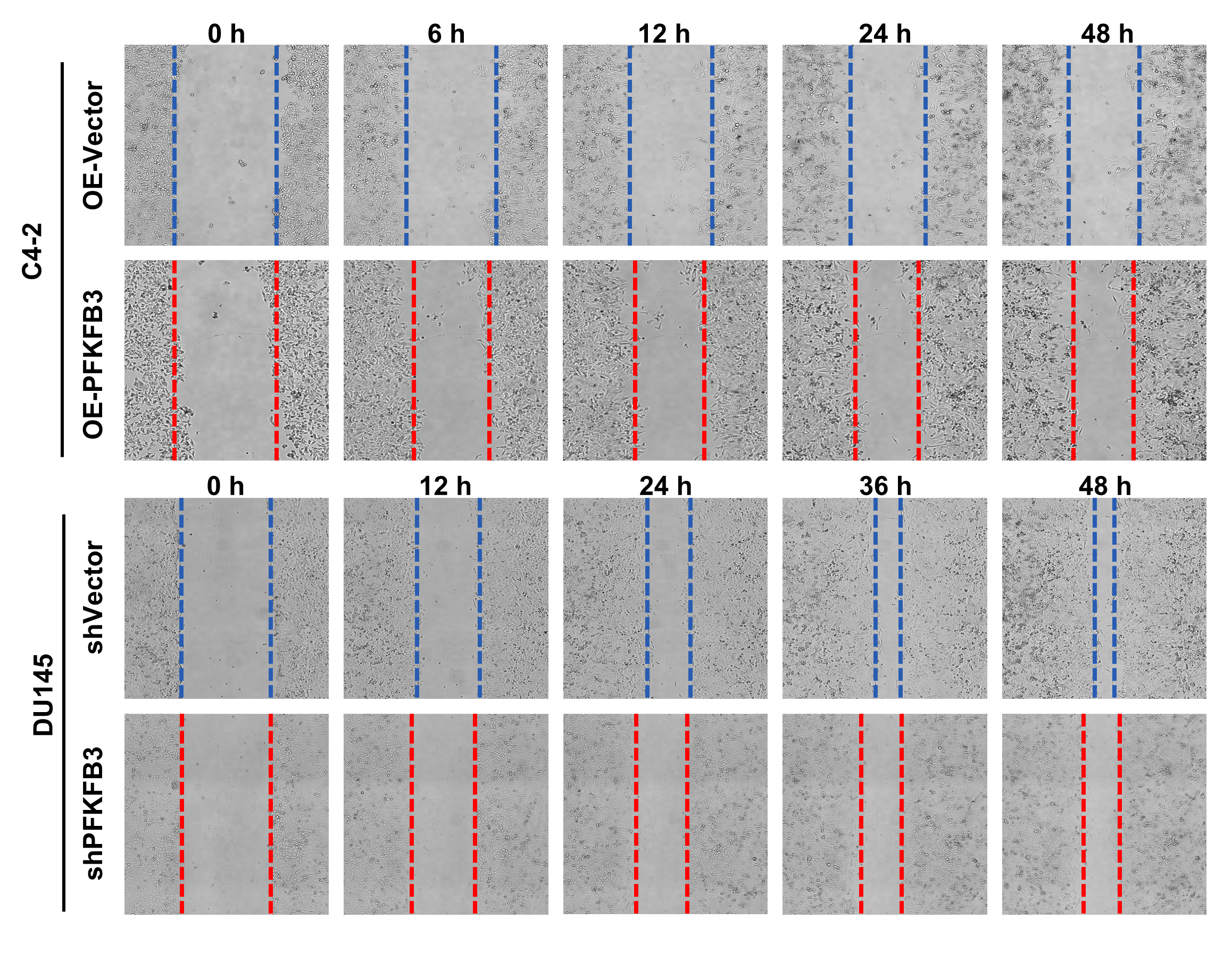

Supplement: Supplementary file 6 — Supplementary figureS5 [file 41419_2025_8089_MOESM6_ESM.png]

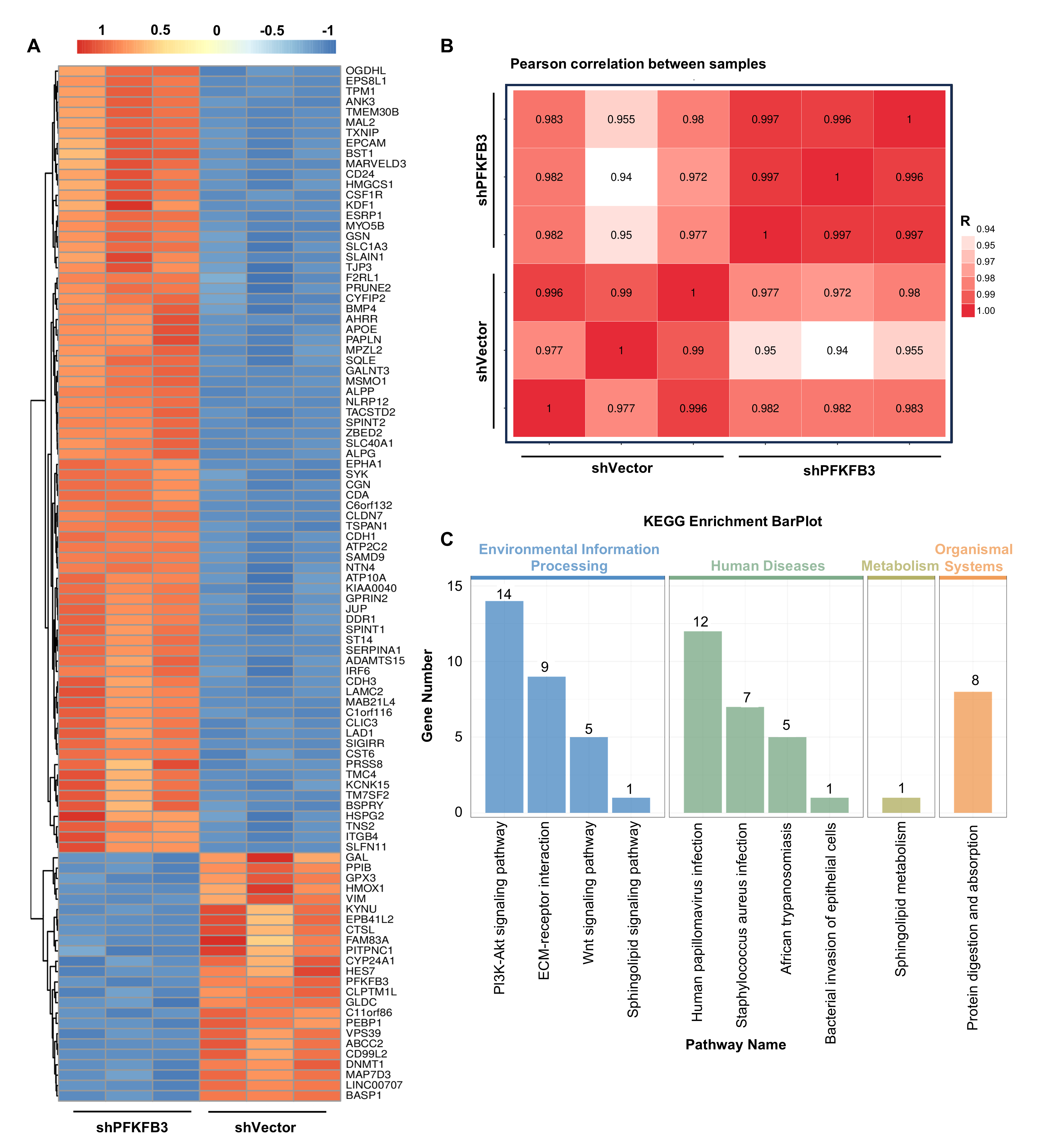

Supplement: Supplementary file 7 — Supplementary figureS6 [file 41419_2025_8089_MOESM7_ESM.png]

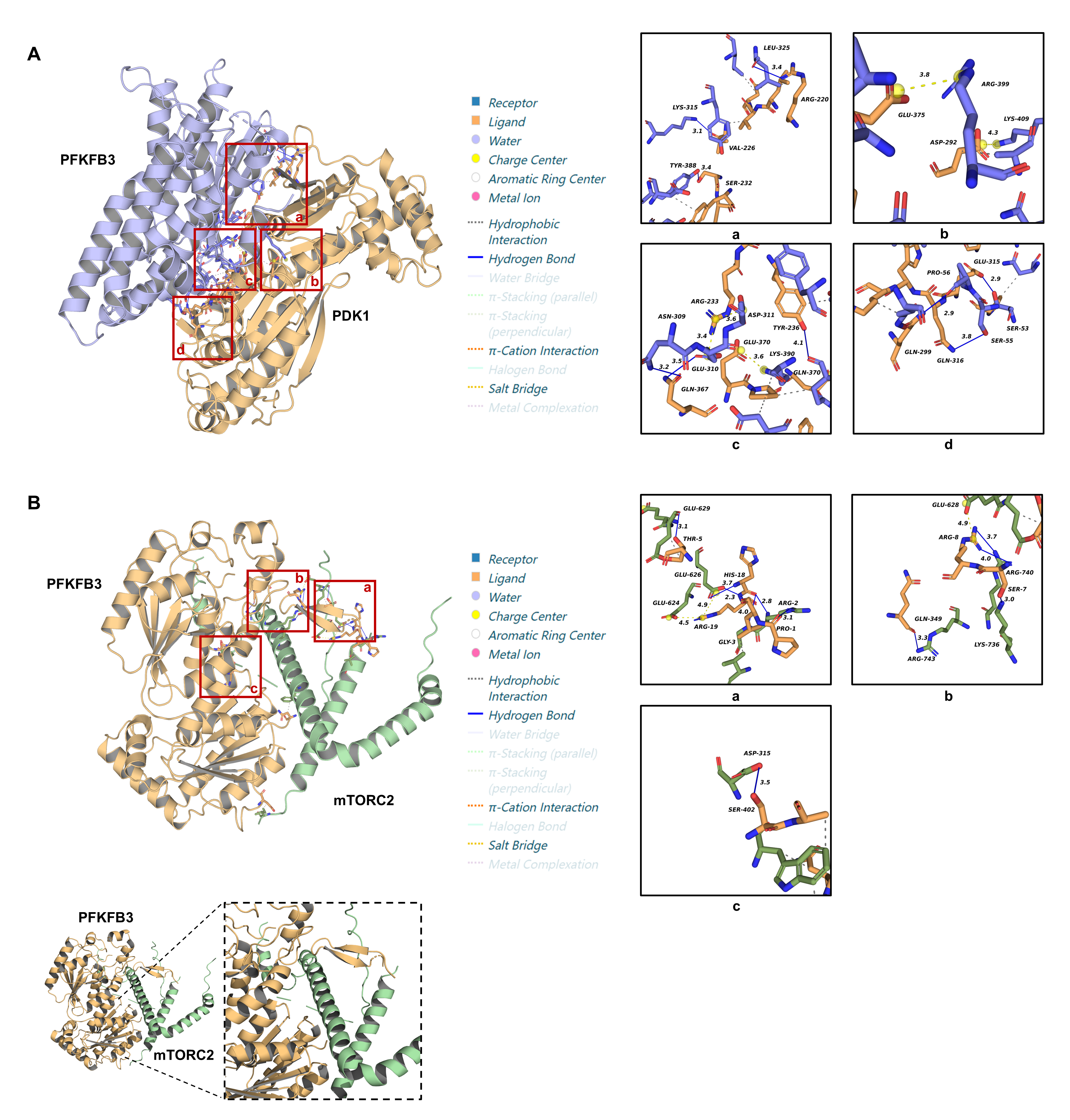

Supplement: Supplementary file 8 — Supplementary figureS7 [file 41419_2025_8089_MOESM8_ESM.png]

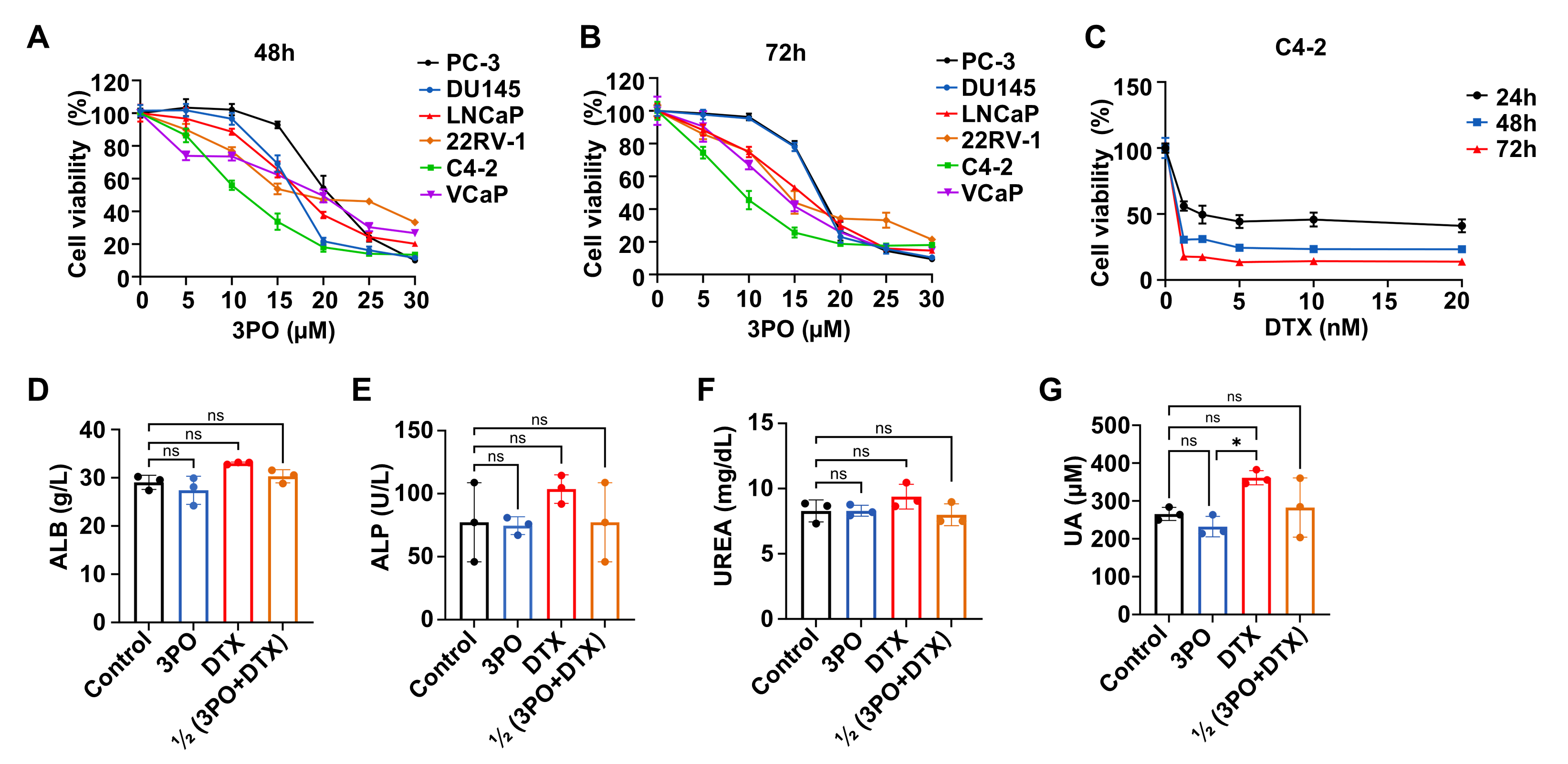

Supplement: Supplementary file 9 — Supplementary figureS8 [file 41419_2025_8089_MOESM9_ESM.png]
